# Low hepatic artery blood flow mediates NET extravasation through the regulation of PIEZO1/SRC signaling to induce biliary complications after liver transplantation

**DOI:** 10.7150/thno.99514

**Published:** 2024-10-14

**Authors:** Hongqiang Yu, Yujun Zhang, Ling Shuai, Cong Peng, Changchun Zhao, Yan Jiang, Ling Yao, Jiejuan Lai, Zhiyu Chen, Leida Zhang, Xiang Xiong, Xiaojun Wang

**Affiliations:** 1 Key Laboratory of Hepatobiliary and Pancreatic Surgery, Institute of Hepatobiliary Surgery, Southwest Hospital, Third Military Medical University (Army Medical University), Chongqing, China 400038, China.; 2 Department of General Surgery, Traditional Chinese Medicine Hospital, Chongqing, China 400015, China.

**Keywords:** Biliary complications, Liver transplantation, Neutrophil extracellular trap, Hepatic artery blood flow, PIEZO1

## Abstract

**Rationale:** Biliary complications after liver transplantation persistently affect patient prognosis and graft survival. Neutrophil-mediated immune injury is an important factor leading to biliary injury. However, the mechanism by which neutrophils reach the periphery of the bile duct and further mediate bile duct injury is not fully understood.

**Methods:** First, we obtained hepatic tissue samples from grafted rats subjected to warm and nonwarm ischemic injury. We constructed a protein map via proteomics and analyzed the correlations between neutrophil extracellular traps (NETs) and biliary injury. HuCCT1 cells were cocultured with NETs isolated from the peripheral blood of grafted rats in vitro to evaluate the role of NETs in bile duct injury. Next, we assessed NET extravasation through the PIEZO1/SRC pathway in liver samples from rats with liver grafts via proteomic analysis, immunohistochemical staining and immunofluorescence. Finally, we evaluated the correlations between hepatic arterial blood flow and the PIEZO1/SRC pathway in a liver graft model.

**Results:** The results revealed a close correlation between NET formation by activated neutrophils and bile duct injury. Low hepatic arterial blood flow leads to NET extravasation through the activation of the mechanosensitive ion channel PIEZO1 and its downstream signaling events, including phosphorylation of tyrosine kinases sarcoma (SRC) protein. The extravasated NETs accumulate around the bile ducts and subsequently mediate biliary cell apoptosis. Verapamil was further used to increase hepatic artery blood flow to inhibit the PIEZO1/SRC axis, which reduced bile duct injury caused by extravasated NETs.

**Conclusions:** Suppressing NET extravasation by increasing hepatic arterial blood flow is a potential strategy for the treatment of biliary complications after liver transplantation.

## Introduction

Liver transplantation is the only effective treatment for end-stage liver disease and has been widely used worldwide 1, 2. However, serious biliary complications still occur in 5% to 20% of patients post-surgery, resulting in the need for secondary liver transplantation or death 3, 4. To date, the major risk factors for biliary complications, such as the surgical technique, antirejection drugs, postoperative infections and cold ischemic storage of the donor liver, have been effectively addressed 3. The use of marginal donor livers has increased due to the increasing demand for donor livers 5, and livers donated after cardiac death (DCD) are currently the main source of donor livers 2. These DCD donor livers are more sensitive to warm ischemia, increasing the risk of biliary complications after liver transplantation to 39% 1, 6. Warm ischemic processes can lead to biliary injury, and the subsequent serious biliary complications after liver transplantation have not been addressed. Therefore, investigating the mechanism of bile duct injury after liver transplantation with warm ischemic donors is important for identifying potential therapeutic targets.

Biliary injury in liver transplantation is caused mainly by oxidative stress injury and immune injury 7, 8. Therefore, researchers have attempted to identify relevant molecular targets to safely, effectively and economically address biliary problems. The pathological process of biliary complications involves multiple cells and complex pathways. Therefore, use of a single molecular target cannot effectively regulate biliary injury. Activated neutrophils are the main cells involved in immune injury and are associated with postoperative apoptosis of liver parenchymal and endothelial cells, leading to complications such as graft dysfunction and acute rejection 9, 10. Histopathological analysis revealed that many lymphocytes aggregated abnormally in damaged liver tissues, even around the bile duct. Neutrophil extracellular traps (NETs) are formed by neutrophil activation and account for the majority of lymphocytes that aggregate at damaged sites. This specific phenomenon and the underlying mechanism need to be further investigated.

Low hepatic artery blood flow is caused by morphological changes in endothelial cells, which cause sustained metabolic disorders of the liver and result in excess reactive oxygen species during liver injury 11, 12. Oxidative stress-induced cells overexpress complement C3 and C5 to activate blood neutrophils 13, 14. Activated neutrophils bind to intercellular adhesion molecule 1 (ICAM-1) on damaged endothelial cells to promote their migration to the damaged site 15, 16. Neutrophils infiltrate the gap of the vasculature to the site of damage via the endothelial cell cation channel PIEZO1 and its downstream tyrosine kinase sarcoma (SRC) and protein tyrosine kinase 2 (PYK2) signaling pathway 17. The pressure change caused by low blood flow increases PIEZO1 protein expression 18, 19. A previous report showed that increasing hepatic artery flow velocity through pharmacological intervention reduced liver injury after liver transplantation 20. Whether blood flow changes can modulate NET extravasation to alleviate biliary injury needs to be verified.

Therefore, we explored the following two aspects via a warm ischemic liver transplantation rat model: the relationship between NET and bile duct injury after hepatic grafting and the specific mechanism involved. Thus, pharmacological intervention is feasible for modulating NET extravasation to ameliorate bile duct injury after liver transplantation.

## Materials and methods

### Experimental animals

Male Sprague‒Dawley (SD) rats (200-220 g) (SJA Animal Laboratory, Hunan, China) were used for all experiments. The animals were maintained on a standard laboratory rodent chow diet and in a specific feeding environment. All experiments were approved by the animal ethics committee (AMUWEC20232992).

### Statistical analysis

R (R 4.1.2) and R-Studio (v7.2 Build 153957) software were used to analyze the data and create statistical charts, and the data are presented as the means ± standard errors. For data processing, the R package MSstats (v4.1.1) was used for data normalization and analysis of differentially expressed proteins. Kyoto Encyclopedia of Genes and Genomes (KEGG) and Gene Ontology (GO) analyses were implemented via the R package clusterProfiler (v3.18.1) with the Enricher function. Gene set enrichment analysis (GSEA) was performed via the R package GSVA (v3.18.2) with the GSVA function and GSEA method. Proteins with a fold change value greater than 2 and a p value less than 0.05 were defined as differentially expressed proteins. All the analyses were performed with R software. Comparisons between 2 groups were performed with a 2-tailed unpaired t test. The correlations between the number of MPO-positive cells and biliary injury scores were determined via Pearson's correlation coefficient. Overall survival of recipient post-surgery was analyzed via the log-rank test and the Kaplan-Meier method. p ≤ 0.05 was defined as statistically significant. Area determination was performed via an imaging system (Olympus, Germany) and FIJI software (ImageJ, National Institutes of Health). Adobe illustrator software (Adobe, USA) was used.

Other materials and methods are described in the supplemental materials and methods.

## Results

### Hepatic arterial blood flow affected by warm ischemic injury and is critically involved in biliary injury

**Figure 1:**
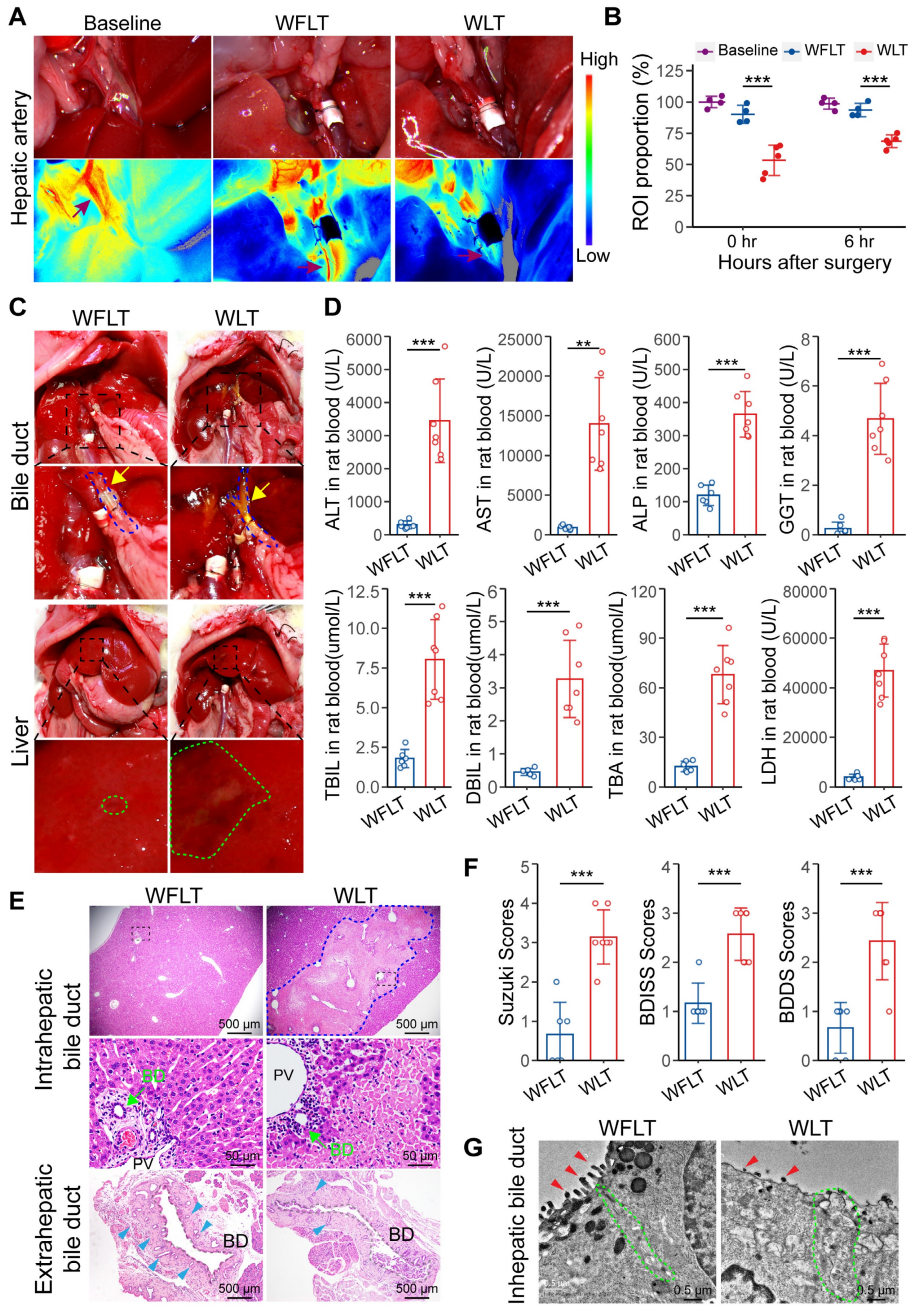
** Hepatic arterial blood flow is critically involved in biliary injury. (A)** Representative laser speckle images of hepatic arterial blood flow at 0 h post-surgery. **(B)** Related region of interest (ROI; wine red arrows in image A) showing the proportions of hepatic arterial blood flow in the baseline group (n = 4), WFLT group (n = 4) and WLT group (n = 5). **(C)** Representative images of liver anatomy in the extrahepatic bile duct and liver surface at 6 h post-surgery. The blue dashed line and yellow arrowheads indicate the blue outline of the bile ducts and extrahepatic bile leakage, respectively. The green outline indicates injured plaques. **(D)** Analyses of clinical parameters of hepatobiliary function in the peripheral blood of the WFLT (n = 6) and WLT (n = 7) groups at 6 h. **(E)** Representative H&E image of bile ducts at 6 h. The blue dashed outline indicates the area of hepatocellular necrosis, and the green and blue teal arrowheads show the bile duct and peribiliary glands, respectively. Scale bars: 500 µm and 50 µm. **(F)** Injury scores of the hepatobiliary duct according to the Suzuki S score, BDISS and BDDS. **(G)** TEM images of the intrahepatic bile duct. The green dashed outlines show areas of cell‒cell junctions, and the red arrowheads indicate scattered MVs. Scale bar: 0.5 μm. BD, bile duct; PV, portal vein. Mean values ± SD. **p < 0.01; ***p < 0.001.

During our experiments, we detected changes in the hepatic arterial blood flow of the rats after liver transplantation from warm ischemic donors. Therefore, the changes in hepatic arterial blood flow in the normal rats (baseline group), the rats with warm ischemia-free liver transplant (WFLT group), and the rats with warm ischemic liver transplant (WLT group) were examined via a laser speckle imaging system to assess changes in blood flow. Compared with that in the normal rats, hepatic arterial blood flow in the WFLT group at 0 and 6 h was reduced by 9.81% and 6.39%, respectively, and that in the WLT group was reduced by 46.68% and 31.38%, respectively. Hepatic arterial blood flow was significantly lower after warm ischemic liver transplantation than after nonwarm ischemia (Figure 1A-B). Similarly, changes in the intrahepatic blood flow were detected, and the trend was consistent with the changes in hepatic arterial blood flow (Figure S2A-B). The results revealed a significant decrease in hepatic arterial blood flow after warm ischemic liver transplantation.

Anatomical analysis revealed extrahepatic bile leakage in the upper bile duct of the anastomosis and intrahepatic injury plaques at 6 h post-surgery in the warm ischemic liver-transplanted rats but nearly not in the nonwarm ischemic rats (Figure 1C). Serum biochemical examination revealed that alanine aminotransferase (ALT), aspartate aminotransferase (AST) and lactate dehydrogenase (LDH) levels were significantly increased and that alkaline phosphatase (ALP), gamma glutamyltransferase (GGT) and total bilirubin (TBIL) levels were significantly increased in the WLT group (Figure 1D). Moreover, the ALT, AST, LDH and ALP levels were notably greater in the WLT group than in the WFLT group at 7 days. Interestingly, the gradual decrease in the ALP level was reversed on day 5, whereas the ALT, AST, and LDH levels barely increased in the WLT group on day 28 (Figure S2C), which might be related to biliary injury. These results indicated that hepatobiliary function was impaired in the rats with warm ischemic grafts. In addition, histopathological analysis revealed extensive hepatocellular necrosis, bile duct cell damage and a reduction in the number of peribiliary glands in the warm ischemic-transplanted rats (Figure 1E). The degree of hepatobiliary damage was assessed by the Suzuki S, BDISS and BDDS methods, and the mean scores in the WFLT group were 0.6, 1.2 and 0.7 (one grade), respectively. The mean scores in the WLT group were 3.1, 2.6 and 2.4 (three grades), indicating severe damage (Figure 1F). Transmission electron microscopy (TEM) revealed that the number of scattered microvilli (MVs) on the intrahepatic cholangiocyte surface was substantially lower and that the number of intercellular junctions was lower after warm ischemic liver transplantation than after nonwarm ischemia (Figure 1G), indicating severe damage to the bile ducts. TUNEL staining also revealed that hepatocytes and cholangiocytes were severely injured in the WLT group (Figure S2D-F). These results demonstrated that the effect of hepatic arterial blood flow on warm ischemic injury may lead to bile duct damage after liver transplantation.

Furthermore, four-week survival was significantly lower in the WLT group (44.4%, 4/9) than in the WFLT group (Figure S3A). H&E and immunohistochemical staining revealed that the number of CK7-positive cholangiocytes was significantly greater in the intrahepatic tissue of the WLT group (Figure S3B-C), indicating a substantial number of proliferating bile ducts four weeks after surgery. Morphometric analysis of Sirius Red-stained hepatic sections revealed that the positive signal area was increased in the WLT group (Figure S3D). Fibrosis grade, which was determined via the METAVIR scoring system, increased in the rats with warm ischemic grafts (Figure S3E). In general, the low hepatic arterial blood flow caused by warm ischemia significantly reduced rat survival and promoted intrahepatic biliary proliferation and fibrosis.

### Construction of a protein map of the rat liver transplant model via proteomics

**Figure 2:**
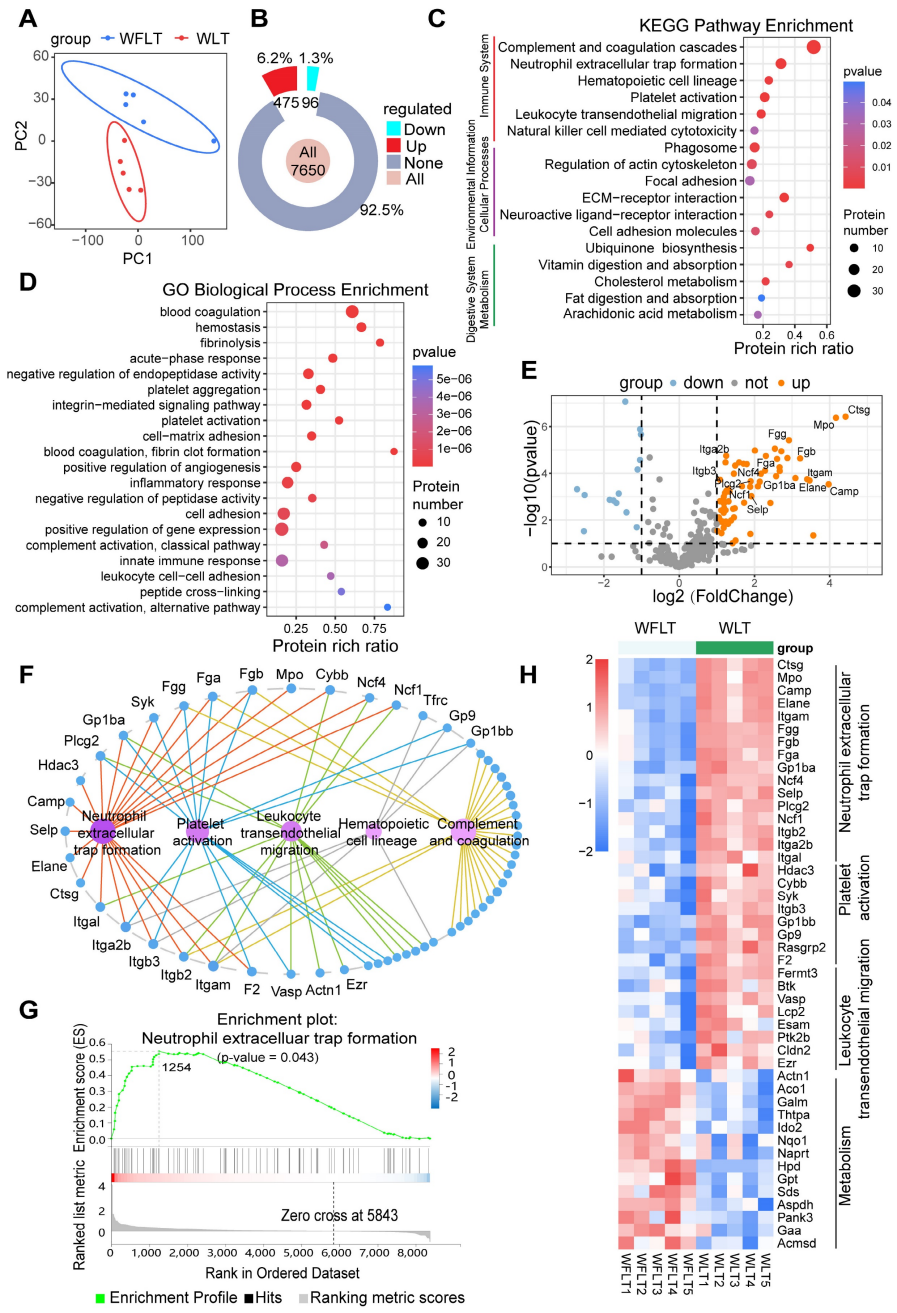
** Proteomic profiling in the rat liver transplant model. (A)** PCA plots of samples from the WFLT and WLT groups. **(B)** Numbers and percentages of upregulated (red), downregulated (cyan) and unchanged (gray) genes between the two groups. **(C)** KEGG and **(D)** GO enrichment plots of differentially expressed proteins (absolute log2-fold change value > 2, p value < 0.05) in the liver tissues. **(E)** Volcano plot of differentially expressed proteins involved in NET formation, platelet activation, complement and coagulation cascades, leukocyte transendothelial migration and metabolic pathways. **(F)** Connected network image of pathways and proteins in immune-related pathways. The nodes in magenta indicate differential pathways, and the nodes in blue indicate differentially expressed proteins. **(G)** GSEA image of the NET formation pathway. **(H)** Heatmap of pathway-related differentially expressed proteins (p values < 0.05); red and blue color scales indicate upregulated and downregulated proteins, respectively.

To further explore the mechanisms by which warm ischemia affects hepatic arterial blood flow, leading to bile duct injury after liver transplantation, we used data independent acquisition (DIA) proteomics to detect changes in liver tissue proteins at 6 h after transplantation and construct protein profiles. Principal component analysis (PCA) revealed that the WFLT and WLT groups presented notably different distributions (Figure 2A). The difference in the median coefficient of variation (CV) was 9.62% (< 10%) between the two groups (Figure S4A-B), indicating that the protein quantification of liver tissues was reliable. Overall, a total of 7650 proteins were detected, and the number of differentially expressed proteins (absolute log2-fold change value > 2 and p value < 0.05) between the two groups was 571 (7.5%), of which 475 (6.2%) proteins were upregulated and 96 (1.3%) were downregulated (Figures 2B, S4C).

Kyoto Encyclopedia of Genes and Genomes (KEGG) functional classification revealed that the differentially expressed proteins were most enriched in the immune system, with 97 (17%) differentially expressed proteins (Figure S4D). Furthermore, KEGG pathway enrichment analysis identified pathways closely related to the immune system, such as pathways related to complement and coagulation cascades, NET formation, platelet activation and leukocyte transendothelial migration (Figure 2C). Gene Ontology (GO) enrichment analysis of biological processes revealed that the differentially expressed proteins were enriched mainly in blood coagulation, fibrinolysis, platelet activation and aggregation, the inflammatory response, and cell adhesion (Figure 2D). The KEGG and GO results revealed that biliary injury may be linked to NET formation, complementary and coagulation processes, platelet activation, and leukocyte transendothelial migration.

A volcano plot of the differentially expressed proteins involved in NET formation, complement and coagulation cascades, platelet activation, leukocyte transendothelial migration and metabolism revealed that the expression of myeloperoxidase (MPO), neutrophil elastase 2 (NE), integrin beta 2 (Itgb2), integrin alpha L (Itgal), fibrinogen alpha (Fga), glycoprotein Ib platelet subunit alpha (GPIba) and other proteins was significantly upregulated (Figure 2E). Among these proteins, Itgb2 and Itgal are involved in NET formation and the leukocyte transendothelial migration pathway, and Fga and GPIba are involved in NET formation and the platelet activation pathway (Figure 2F, S4E-F). Gene Set Enrichment Analysis (GSEA) and a heatmap revealed that the differentially expressed proteins were significantly enriched in the NET formation pathway (p = 0.043) (Figure 2G-H, S4G), suggesting that NET may be strongly associated with bile duct injury.

### NET leads to bile duct injury after warm ischemic liver transplantation

**Figure 3:**
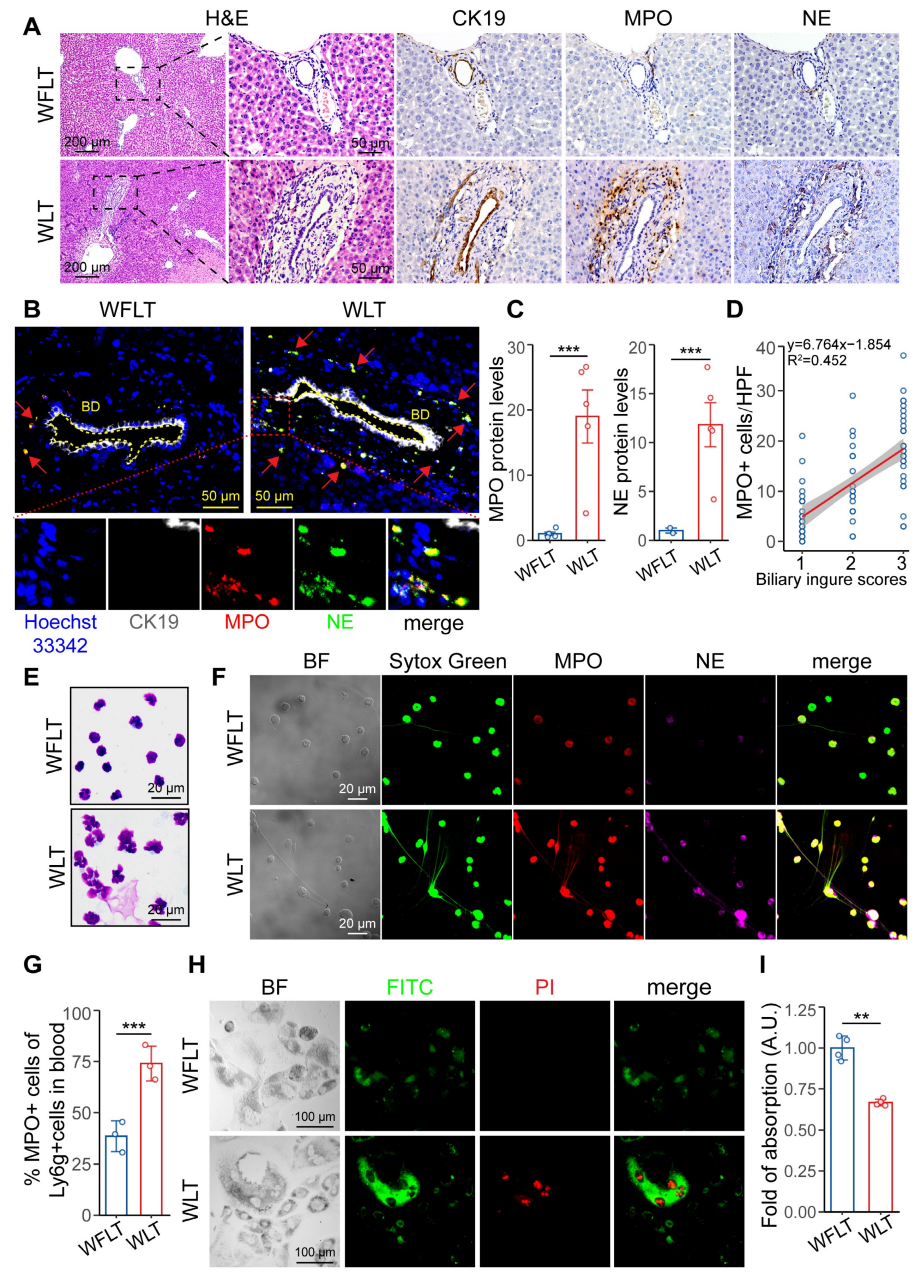
** NET formation is associated with biliary damage after warm ischemic liver transplantation. (A)** H&E and immunohistochemical staining images of grafted liver tissues. CK19 labeled the intrahepatic bile ducts, and MPO and NE labeled the NETs. Scale bars: 200 µm and 50 µm. **(B)** Immunofluorescence of liver tissue samples from intrahepatic bile ducts (indicated by CK19) and NETs (indicated by MPO and NE). The blue indicates the cellular nucleus, and the red arrows indicate the coexpressed area. Scale bar: 50 µm. **(C)** MPO and NE protein levels in liver tissue were determined via proteomics. **(D)** Line correlation plot between biliary injury scores and the number of MPO+ cells around the bile duct. **(E)** Giemsa-stained images of activated neutrophil cells isolated from peripheral blood at 6 h post-surgery. Scale bar: 20 µm. **(F)** Immunofluorescence images of NET formation in peripheral blood at 6 h after surgery. Scale bar: 20 µm. **(G)** quantification (in image figure S6A) of peripheral blood Ly6G+ and MPO+ cells in post-surgery rats. **(H)** Immunofluorescence images of HuCCT1 cell apoptosis induced by coculture with NETs for 24 h via an apoptosis kit. BF, bright field. Scale bar: 100 µm. **(I)** The viability of HuCCT1 cells cocultured with NETs for 24 h was measured via the MTT assay. BD, bile duct. Mean values ± SD. **p < 0.01; ***p < 0.001.

Immunohistochemical staining and histopathological analysis revealed that MPO- and NE-specific labeled NETs were enriched around the injured bile ducts (CK19-specific labeling) (Figure 3A). Many NETs accumulated abnormally in the bile ducts and surrounding tissues (Figure 3B). Proteomic analysis was used to quantify the MPO and NE protein expression levels and revealed that the expression of MPO and NE in the liver tissues of the WLT group was 18.9- and 11.8-fold greater than that in the WFLT group, respectively (Figure 3C). A linear correlation plot revealed that the biliary injury scores were significantly positively related (R^2^ = 0.452) to the number of MPO+ cells around the bile duct according to the immunohistochemical staining images (Figure 3D), suggesting a close correlation between the NET level and the degree of bile duct injury. To eliminate the possibility of macrophages forming extracellular traps and expressing the MPO protein, we assessed the MPO protein levels in the macrophages of grafted rat liver tissue samples. Immunofluorescence staining and colocalization analysis revealed that MPO-specific labeled NETs were colocalized mainly on neutrophils (Ly6G-specific labeling, r = 0.69), enriched around the injured bile ducts, and rarely colocalized on macrophages (F4/80-specific labeling, absolute r = 0.17) in the WLT group (Figure S5A-D), indicated that NET formation is mainly generated by neutrophils at 6 h after liver transplantation.

To demonstrate that NETs may lead to biliary cell apoptosis, we isolated activated neutrophils from the peripheral blood of rats after liver transplantation and stained them with Giemsa. The staining results revealed clear nuclear deformity and outward cytoplasmic extensions of the neutrophils (Figure 3E), indicating the presence of NETs in the peripheral blood of the warm-ischemic transplanted rats. Moreover, immunofluorescence revealed that the fluorescence intensity and quantity of MPO, NE and filamentous DNA in the WLT group were significantly greater than those in the WFLT group (Figure 3F). Quantitative flow cytometric analysis further revealed that the percentage of peripheral blood neutrophils activated to form NETs in the warm ischemia-injured grafted rats was 74.03%, which was significantly greater than that in the nonwarm ischemic rats (38.63%) (Figure 3G, S6A). The enzyme-linked immunosorbent assay (ELISA) and double-stranded DNA (dsDNA) detected results revealed that the levels of MPO and dsDNA were significantly greater in the peripheral blood of the WLT group than in the WFLT group (Figure S6B). To demonstrate the relationship between NET and biliary cells, we used an apoptosis kit to measure the level of HuCCT1 apoptosis induced by NETs, and more apoptotic signals were observed in biliary cells cocultured with NETs isolated from the blood of the warm ischemic-transplanted rats (Figure 3H). Moreover, the viability of the HuCCT1 cells cocultured with NETs was 0.33-fold lower in the WLT group than in the WFLT group (Figure 3I), demonstrating that NETs can promote bile duct cell apoptosis. The results indicated that a large amount of aggregated NETs increased biliary damage after warm ischemic liver transplantation.

In addition, to further verify the bile duct injury caused by NETs in grafted rats, we used an NE inhibitor (sivelestat) to reduce NET formation through the inhibition of NE protein function in neutrophils. Treatment of recipients with 50 mg/kg sivelestat via intraperitoneal injection postoperatively reduced intrahepatic NET formation in the warm ischemic liver-transplanted rats (the sivelestat group). Autopsy revealed that extrahepatic bile leakage and hepatic injury plaques were reduced at 6 h after surgery in the sivelestat-treated rats (Figure S7A-B). Serum biochemical tests revealed that the hepatobiliary function of the sivelestat-treated rats was significantly better than that of the untreated rats (Figure S7C-D). Furthermore, the H&E and immunohistochemical staining revealed that the distribution of MPO and NE around the bile ducts and the number of positive cells were significantly reduced in the liver tissues of the sivelestat-treated group (Figure S7E-F). TUNEL staining revealed that the number of cholangiocyte- and hepatocyte-positive cells was significantly decreased in the liver tissues of the rats treated with sivelestat (Figure 7G-H). The results showed that NET formation inhibited by sivelestat could alleviate hepatic bile duct injury after warm ischemic liver transplantation, suggesting that NET formation plays a crucial role in biliary injury.

Complement C3 and C5 have been shown to induce neutrophil activation to form NETs 13, 14. The increased protein levels of C3 and C5 were confirmed by immunofluorescence (Figure S8A-B) and proteomic quantification (Figure S8C) in rat liver tissues subjected to warm ischemia injury. Western blotting experiments revealed that the C3 and C5 levels in the liver sinusoidal endothelial cells were visibly increased in the WLT group (Figure S8D). C3 and C5 are cleaved to form C3a and C5a which can bind to C3a and C5a receptors to activate neutrophils 21-23. The C3a and C5a levels were significantly increased in the rat peripheral blood of WLT group via ELISA (Figure S8E). Recombinant C3a- or C5a-stimulated neutrophils that formed NETs were also observed in vitro*.* Fluorescence staining image showed that the NET formation was significantly elevated when neutrophils were treated with recombinant C3a or C5a (Figure S8F-G). Western blotting result revealed that the levels of MPO and NE proteins were positively related with the level of C3 and C5 protein in vitro and in vivo (Figure S8H-K). the phenomenon suggested that the complement system (especially C3 and C5) was closely related to NET formation in our rat liver graft model.

### Overactivation of the PIEZO1/SRC axis by low hepatic arterial blood flow promotes the extravasation of NETs

**Figure 4:**
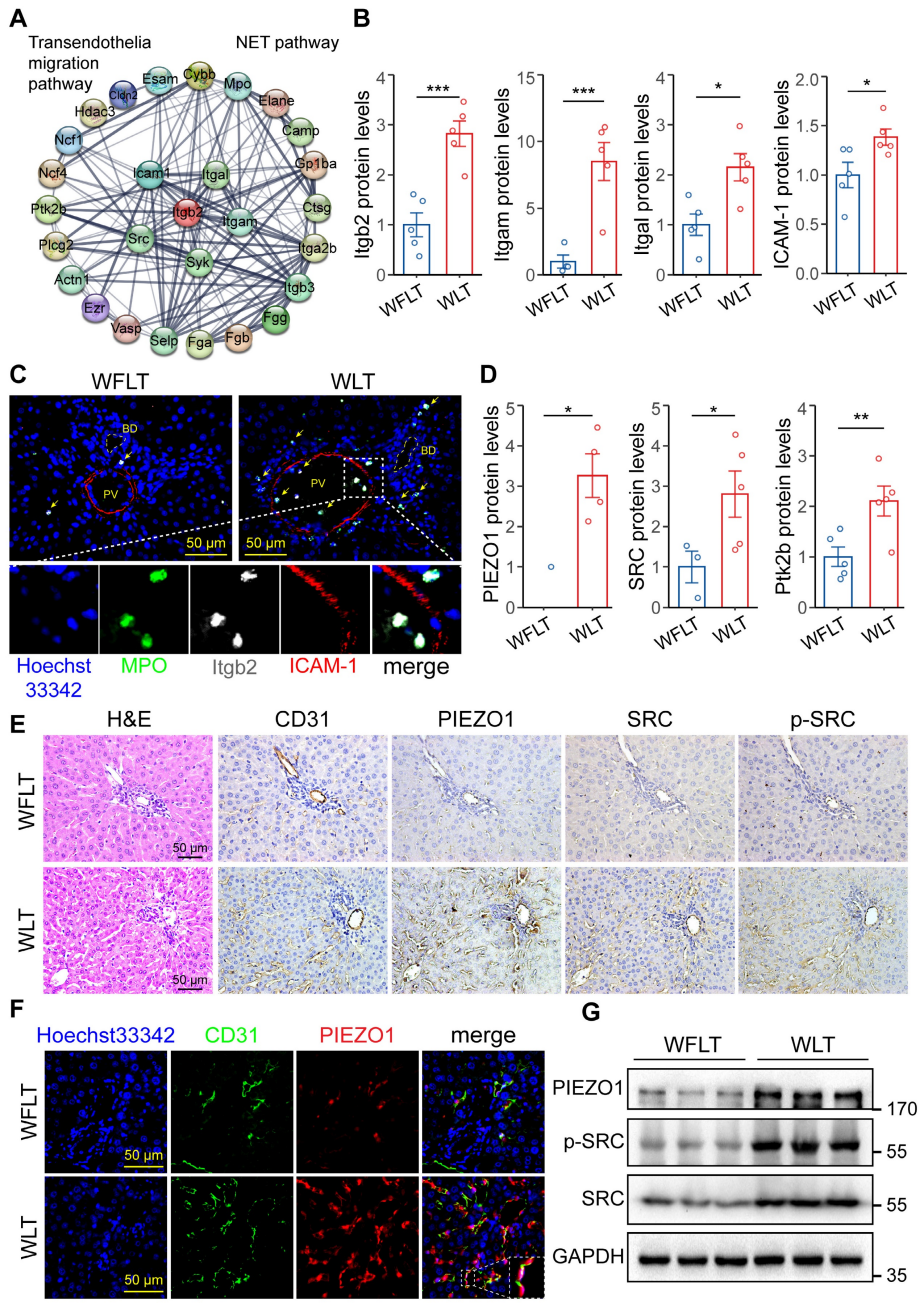
** Activation of the PIEZO1/SRC axis is involved in NET extravasation. (A)** Protein‒protein interaction (PPI) network of NET formation and the transendothelial migration pathway. **(B)** Expression levels of Itgal, Itgam, Itgb2 and ICAM-1 determined via proteomic analysis. **(C)** Immunofluorescence image of postoperative hepatic tissue. MPO-labeled NETs and ICAM-1-labeled endothelial cells. Blue indicates cellular nuclei, and yellow arrows indicate coexpression. **(D)** Protein levels of PIEZO1, SRC and Ptk2b in liver tissues determined by proteomic analysis. **(E)** Images of H&E and immunohistochemical staining for CD31, PIEZO1, SRC and p-SRC in hepatic tissue sections from liver graft samples. The CD31 and PIEZO1 proteins are expressed in vascular endothelial cells. **(F)** Immunofluorescence staining of the CD31 and PIEZO1 proteins in liver tissue samples. An anti-CD31 antibody was used to identify endothelial cells. The blue-labeled nucleus and white dashed line indicate the magnified coexpressed area. **(G)** The protein levels of GPADH, PIEZO1, SRC and p-SRC in liver tissues were determined by Western blotting. Scale bar: 50 µm. BD, bile duct; PV, portal vein. Mean values ± SD. *p < 0.05; **p < 0.01; ***p < 0.001.

We investigated how NETs in the blood accumulate around bile ducts and lead to bile duct damage. A protein‒protein interaction (PPI) network of the differentially expressed proteins related to NET formation and the transendothelial migration pathway was constructed, and the results revealed that the proteins Itgb2, Itga1, Itgam, ICAM-1 and SRC were important nodes (Figure 4A). Proteomic quantification showed that the protein expression levels of Itgb2, Itgam, Itgal and ICAM-1 in the warm ischemic graft liver tissues were increased by 1.82-, 7.49-, 1.15- and 0.38-fold, respectively, compared with those in the nonwarm ischemic tissues (Figure 4B). The association of Itgb2 with endothelial ICAM-1 has been reported to induce cellular extravasation 15, 16. Immunofluorescence was used to demonstrate Itgb2-mediated NET vascular extravasation. The results revealed that the colocalization of the MPO-labeled NETs and Itgb2 proteins, which are closely related to ICAM-1-labeled endothelial cells, was significantly increased in the warm ischemic graft hepatic tissues (Figure 4C). Immunofluorescence revealed that MPO-labeled NETs adhered to the vascular endothelial cell layer and crossed the α-SMA-labeled vascular smooth muscle layer before being distributed around the bile ducts (Figure S9A-B). Studies have reported that mechanical stress and Itgb2-ICAM-1 clustering synergistically activate PIEZO1 and its downstream SRC/PYK2 signaling to promote cellular extravasation 17, 24. Therefore, to determine whether altered hepatic blood flow can promote NET extravasation, we performed proteomic quantification, which revealed that the protein levels of PIEZO1, SRC and protein-tyrosine kinase 2-beta (Ptk2b) were significantly increased in the warm ischemic transplanted liver tissues (Figure 4D). These results indicated that the low hepatic arterial blood flow caused by warm ischemia could activate the PIEZO1 protein and its downstream signaling via mechanical stress. Immunohistochemistry and immunofluorescence assays revealed increased signaling of PIEZO1 and the downstream proteins SRC and phosphorylated SRC (p-SRC) on vascular endothelial cells in the warm-ischemic graft liver tissues (Figure 4E-F). Moreover, the intrahepatic PIEZO1, SRC and p-SRC protein expression levels were increased in the warm ischemic-transplanted hepatic tissues, as shown by Western blotting (Figure 4G). These results demonstrated that low hepatic arterial blood flow synergized with ICAM-1 clustering to promote NET extravasation through the activation of PIEZO1 and downstream SRC signaling after warm ischemic liver transplantation.

### Increased hepatic arterial blood flow inhibits PIEZO1/SRC signaling to reduce NET vascular extravasation

**Figure 5:**
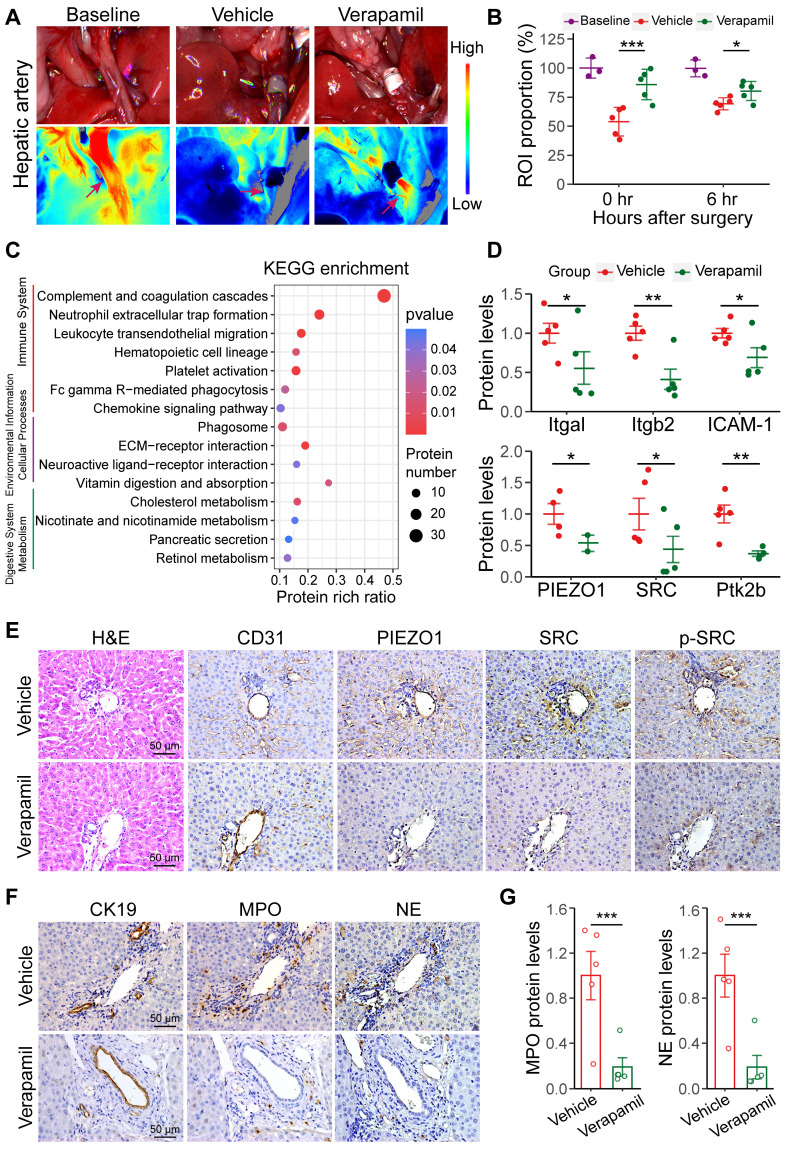
** Increased hepatic arterial blood flow is closely involved in NET vascular extravasation. (A)** Representative laser speckle images of hepatic arterial blood flow at 0.25 h post-surgery. **(B)** ROI (wine red arrows in image A) showing the proportions of hepatic arterial blood flow in baseline group (n = 3), vehicle group (n = 5) and verapamil group (n = 5).** (C)** KEGG enrichment plot of differentially expressed proteins (absolute log2-fold change > 2, p value < 0.05) in the liver tissue. **(D)** The protein levels of Itgal, Itgb2, ICAM-1, PIEZO1, SRC and Ptk2b in liver tissue were determined via proteomics. **(E)** H&E and immunohistochemistry staining images of grafted liver tissues. CD31 labeled the endothelial cells. **(F)** Immunohistochemical staining images of liver tissue samples from intrahepatic bile ducts (indicated by CK19) and NET (indicated by MPO and NE). **(G)** MPO and NE protein levels were detected via proteomics. Scale bar: 50 µm. Mean values ± SD. *p < 0.05; **p < 0.01; ***p < 0.001.

Verapamil was used to increase hepatic artery blood flow to inhibit NET extravasation by preventing the activation of the PIEZO1/SRC axis after warm ischemia-induced liver transplantation. Pretreatment of recipients with 1.5 mg/kg verapamil administered via preoperative gavage increased hepatic arterial blood flow in the warm ischemic liver-transplanted rats (the verapamil group). Laser speckle imaging at 0.25 and 6 h after liver transplantation revealed that hepatic arterial blood flow increased by 31.99% and 11.01%, respectively, in the verapamil-treated rats compared with that in the treatment-free rats (Figure 5A-B); similarly, intrahepatic blood flow increased by 38.44% and 16.77%, respectively (Figure S10A-B). The results showed that oral administration of verapamil effectively increased hepatic arterial blood flow into the damaged transplanted livers. The activation of the PIEZO1/SRC pathway and NET formation were analyzed via proteomic profiling after warm ischemic liver transplantation with verapamil pretreatment. The PCA results revealed that the nontreated and verapamil-treated groups presented distinct distributions (Figure S10C). KEGG annotation, volcano mapping and heatmap analyses revealed that the protein levels of MPO, NE, Itgb2, Itgal and other proteins involved in pathways related to graft injury were decreased after verapamil intervention (Figure 5C, S10D-E). Proteomic quantification revealed that the protein levels of Itgam, Itgal, Itgb2 and ICAM-1 were decreased and that the protein expression levels of PIEZO1, SRC and Ptk2b were decreased in the verapamil-pretreated hepatic tissues (Figure 5D). These results indicated that verapamil could affect the levels of proteins involved in extravasation-related signaling. Furthermore, immunohistochemistry revealed that the protein levels of PIEZO1 and downstream SRC and p-SRC in intrahepatic vascular endothelial cells were reduced in the verapamil-pretreated rats (Figure 5E). The western blotting results revealed that the levels of PIEZO1, SRC and p-SRC were significantly reduced in the rat liver sinusoidal endothelial cells pretreated with verapamil in vivo (Figure S10F-G). In addition, the distribution of MPO and NE around the bile ducts and their protein expression levels were reduced in the liver tissues after verapamil treatment (Figure 5F-G). The ELISA and dsDNA detected results revealed that the MPO and dsDNA levels were significantly reduced in the peripheral blood of the rats in the verapamil group (Figure S10H). These results demonstrated that increasing hepatic arterial blood flow via verapamil pretreatment could reduce NET extravasation by inhibiting the PIEZO1/SRC axis.

### Increasing hepatic arterial blood flow reduces bile duct injury after liver transplantation

**Figure 6:**
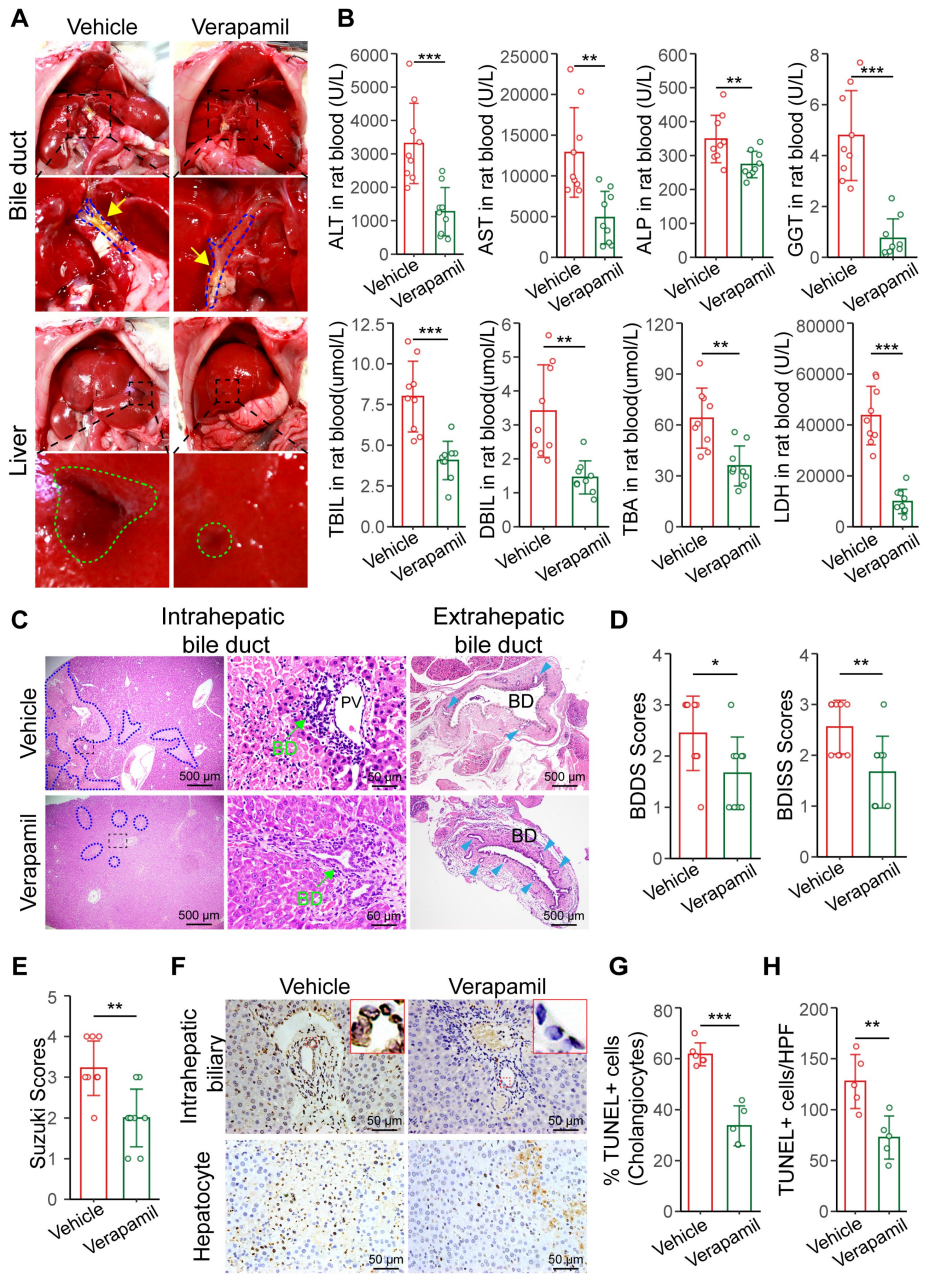
** Increasing hepatic arterial blood flow reduces bile duct injury. (A)** Representative images of liver anatomy in the extrahepatic bile duct and liver surface at 6 h post-surgery. The blue dashed line and yellow arrowheads indicate the blue outline of the bile ducts and extrahepatic bile leakage, respectively. The green outline indicates the injured plaques on liver surface. **(B)** Analyses of clinical parameters of hepatobiliary function in the peripheral blood of the vehicle group (n = 9) and verapamil group (n = 9) at 6 h post-surgery. **(C)** Representative H&E image of intra- and extrahepatic bile ducts at 6 h. The blue dashed outline indicates the area of hepatocellular necrosis, and the green arrowheads indicate the intrahepatic bile duct, and the blue teal arrowheads indicate the peribiliary glands, respectively. Scale bars: 500 µm and 50 µm. The injury scores of the **(D)** intrahepatic and extrahepatic bile ducts and **(E)** hepatocellular region according to the BDISS, BDDS and Suzuki S score, respectively. **(F)** The TUNEL staining images of liver tissue samples from intrahepatic biliary cells and hepatocytes. Scale bar: 50 µm. **(G)** Quantification of TUNEL-positive biliary cell percentages in image F. **(H)** Quantification of TUNEL-positive hepatic cells in image F. Scale bar: 50 µm. BD, bile duct; PV, portal vein. Mean values ± SD. *p < 0.05; **p < 0.01; ***p < 0.001.

Autopsy revealed that extrahepatic bile leakage and hepatic injury plaques were reduced at 6 h post-surgery in the verapamil-treated rats (Figure 6A). Serum biochemical tests revealed that the hepatobiliary function of the verapamil-pretreated rats was significantly better than that of the untreated rats (Figure 6B). ALT, AST, LDH and ALP levels gradually became lower in the verapamil group than in the vehicle group at 28 days (Figure S11A). The results showed that verapamil could alleviate hepatic bile duct injury after warm ischemic liver transplantation. Histopathological analyses revealed that intra- and extrahepatic bile duct injury, the extent of hepatocellular necrosis, and extrahepatic peribiliary gland damage were reduced in the verapamil-pretreated rats (Figure 6C); moreover, the BDISS (from 2.55 to 1.66), BDDS (from 2.44 to 1.66) and Suzuki S score (from 3.2 to 2) were significantly lower (Figure 6D-E). TUNEL staining revealed that the number of cholangiocyte- and hepatocyte-positive cells was significantly decreased in the liver tissues of the rats pretreated with verapamil (Figure 6F-H). These results revealed that increasing hepatic arterial blood flow via verapamil pretreatment significantly alleviated hepatobiliary damage in warm ischemic liver-transplanted rats.

Furthermore, the four-week survival rate was significantly greater (by 36.36%) in the verapamil group than in the vehicle group (Figure S11B). H&E and immunohistochemical staining revealed that the number of CK7-labeled positive bile ducts was significantly lower in the intrahepatic tissue of the verapamil-pretreated grafted rats than in that of the control rats (Figure S11C-D), suggesting that a small number of bile ducts had proliferated four weeks after surgery. Morphometric analysis of Sirius Red-stained hepatic sections revealed that the positive signal area was reduced in the verapamil-pretreated grafted rats (Figure S11E). The fibrosis grade determined via the METAVIR scoring system was significantly lower in the verapamil-pretreated rats than in the non-treated rats (Figure S11F). In general, increasing hepatic arterial blood flow with verapamil significantly increased rat survival and alleviated intrahepatic biliary proliferation and fibrosis.

## Discussion

**Figure 7:**
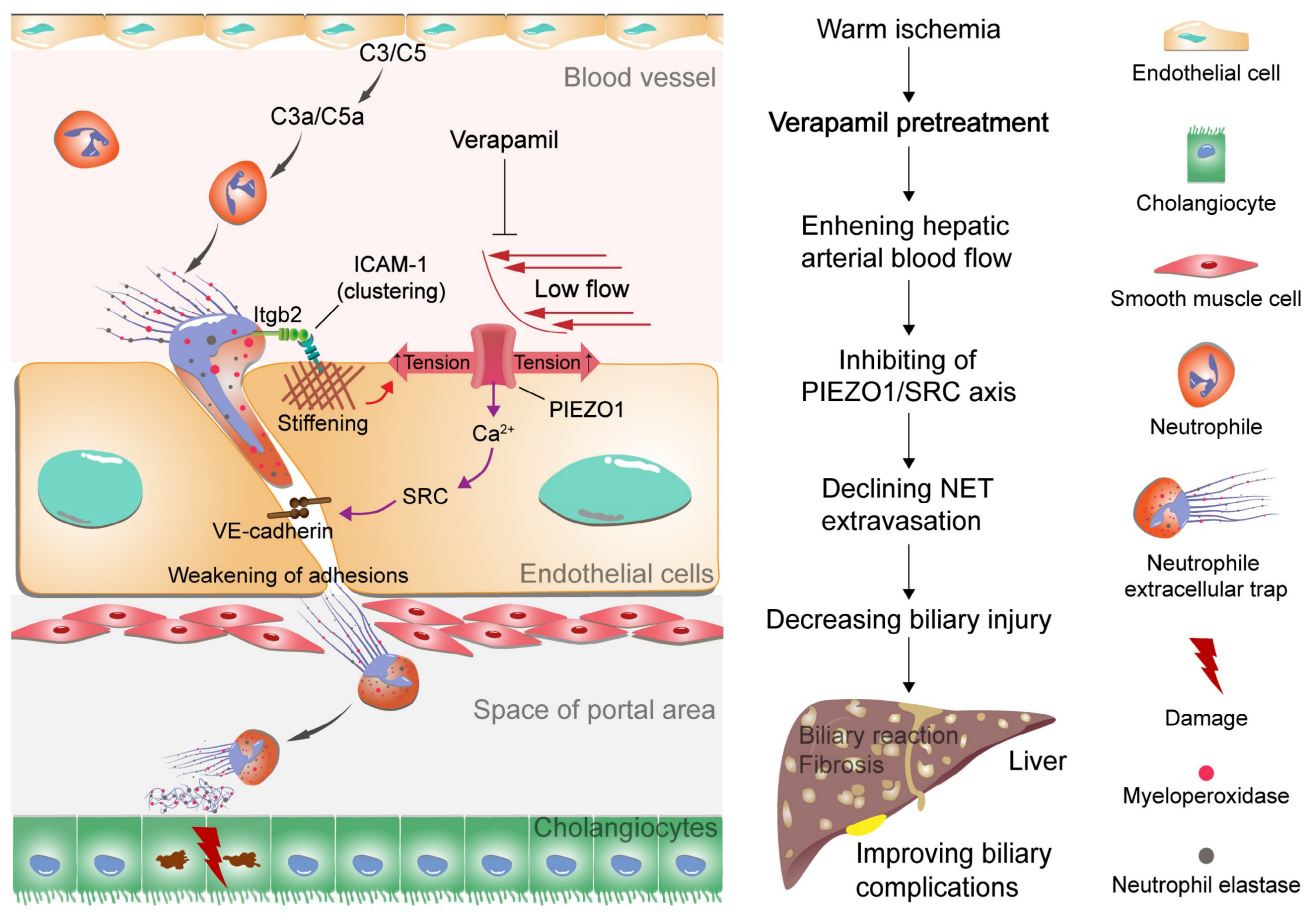
** Proposed model of NET extravasation-related signaling and biliary complications.** Neutrophils are activated by complement C3 and C5 to form NETs. NETs bind to ICAM-1 on endothelial cells via Itgb2 to form ICAM-1 clusters. This clustering synergizes with low hepatic arterial blood flow to activate PIEZO1 to induce downstream signaling events, resulting in weakening of endothelial junctions. An increase in NETs leads to biliary cell apoptosis. Verapamil elevates hepatic arterial blood flow to inhibit NET extravasation and alleviate biliary complications.

Warm ischemic-reperfusion injury is a major risk factor for biliary complications after liver transplantation; however, its pathological mechanisms have not been fully elucidated. In this study, we explored the pathological mechanism by which NET synergize with low arterial blood flow to activate the PIEZO1/SRC axis to mediate vascular extravasation and accumulation around the bile ducts, leading to biliary injury. Moreover, the relationship between low hepatic arterial blood flow and NET vascular extravasation leading to bile duct injury was revealed in warm-ischemic liver-transplanted rats to provide a potential strategy for the clinical treatment of biliary complications after liver transplantation (Figure 7).

Hepatobiliary injury is closely related to changes in hepatic arterial blood flow after liver transplantation 25, 26. Hepatic arterial blood flow, biochemical parameters and pathomorphological structures of the transplanted liver did not significantly change after warm ischemia-free treatment. Warm ischemia-free grafted animals have a greater survival rate. In contrast, hepatic arterial blood flow was significantly reduced after warm ischemia liver transplantation and bile leakage. Furthermore, abnormal levels of biliary function indicators and apoptosis of bile duct cells were found in rats with low hepatic arterial blood flow. The reason for this phenomenon might be that low hepatic arterial blood flow leads to biliary injury. Proteomic profiling was used to identify the possible pathways involved in biliary injury in the graft liver. KEGG enrichment analysis revealed that intrahepatic bile duct injury had the strongest correlation with immune system-related NET formation, platelet activation, and leukocyte transendothelial migration pathways at 6 h after transplantation. Volcano diagrams and heatmaps revealed that the levels of MPO, NE, Itgb2 and other proteins involved in NET formation were abnormally increased, suggesting that NETs plays an important role in the biliary damage after liver transplantation. Proteomic and immunofluorescence assays revealed that the NET level abnormally increased and that NETs were distributed around the damaged bile ducts in warm ischemic livers. Neutrophils were majorly activated through C3a and C5a cleaved by complement C3 and C5 to form NETs in warm ischemic injured grafted model via immunofluorescence, western blotting and ELISA assay. Proteomic and immunofluorescence assays revealed that the NET level abnormally increased and that NETs were distributed around the damaged bile ducts in warm ischemic livers. Moreover, NETs isolated from peripheral blood led to biliary cell apoptosis, indicating that NETs caused biliary injury after liver transplantation. Sivelestat (NE inhibitor) effectively regulated NET formation by inhibiting the function of NE protein to reduce bile duct injury. The phenomenon further proves that bile duct injury was majorly caused by NETs in grafted rats.

To elucidate the process by which NETs in the blood extravasate to the periphery of the intrahepatic bile ducts, we performed protein interaction analysis. The results revealed an increase in the protein levels of Itgb2 and ICAM-1, which are associated with transendothelial permeation. Itgb2 binds to ICAM-1 to form clusters, which promote transendothelial cell extravasation 27, 28. Immunofluorescence experiments revealed that NETs adhered to endothelial cells and extravasated through the vascular barrier to the periphery of bile ducts. Vascular endothelial cells can activate the PIEZO1/SRC/PYK2 axis under mechanical pressure to promote cellular extravasation 17, 18. Proteomic analysis revealed that PIEZO1 protein expression was lower in nonwarm livers. However, the protein expression of PIEZO1, downstream SRC and Ptk2b was significantly increased in the warm ischemic graft livers, indicating that the extravasation pathway was activated in warm ischemic livers. Hepatic blood flow synergizes with NETs to activate the PIEZO1/SRC axis, causing NET extravasation and biliary cellular injury in warm ischemic-transplanted rats.

Furthermore, verapamil was used to increase hepatic arterial blood flow to inhibit NET extravasation. Laser speckle imaging revealed a significant increase in hepatic artery blood flow with verapamil intervention. Proteomics and immunohistochemistry revealed that the protein levels of PIEZO1, SRC and Ptk2b significantly decreased, indicating that an increase in hepatic arterial blood flow inhibited the extravasation-related signaling pathway. Immunohistochemistry revealed that the distribution of NETs around the intrahepatic bile ducts was reduced. These results demonstrated that verapamil inhibited PIEZO1 expression and downstream signaling to reduce NET extravasation. Biochemical tests and histopathological morphometric analysis revealed that verapamil intervention could reduce biliary injury. These findings verified that verapamil could reduce bile duct injury by inhibiting NET extravasation in warm ischemic livers, thereby decreasing biliary complications and increasing survival.

In conclusion, abnormal NET extravasation could promote biliary injury. However, the changes in coagulation pathway-related proteins involved in NET-mediated biliary damage need to be further studied. In conclusion, NET synergized with low hepatic arterial blood flow to activate PIEZO1 and its downstream SRC signaling pathway to promote NET vascular extravasation, which caused biliary injury in warm ischemic-transplanted rats. In contrast, the increase in hepatic arterial blood flow after treatment with verapamil reduced bile duct injury by inhibiting NET extravasation.

## Supplementary Material

Supplementary materials and methods, figures.

## Abbreviations

ALP: alkaline phosphatase
ALT: alanine aminotransferase
AST: aspartate aminotransferase
α-SMA: alpha-smooth muscle actin
BD: bile duct
CK7: cytokeratin 7
CK19: cytokeratin 19
CD31: platelet endothelial cell adhesion molecule 1
C3: complement 3
C5: complement 5
C3a: alpha-chain of C3
C5a: alpha-chain of C5
CV: coefficient of variation
DBIL: direct bilirubin
dsDNA: double-stranded DNA
ELISA: enzyme-linked immunosorbent assay
Fga: fibrinogen alpha
GGT: gamma glutamyltranspeptidase
GPIba: glycoprotein Ib platelet subunit alpha
GO: gene ontology
GSEA: gene set enrichment analysis
H&E: hematoxylin and eosin
HPF: high powered field
ICAM-1: intercellular adhesion molecule 1
Itgal: integrin alpha L
Itgb2: integrin beta 2
IVC: inferior vena cava
KEGG: kyoto encyclopedia of genes and genomes
LDH: lactate dehydrogenase
LPF: low powered field
LSEC: liver sinusoidal endothelial cell
MPO: myeloperoxidase
MVs: microvilli
MTT: methyl tetrazolium
NE: neutrophil elastase 2
NETs: neutrophil extracellular traps
OLT: orthotopic liver transplantation
PCA: principal component analysis
PPI: protein‒protein interaction
p-SRC: phosphorylated tyrosine kinase sarcoma
PV: portal vein
PYK2: protein tyrosine kinase 2
Ptk2b: protein tyrosine kinase 2 beta
SR: sirius red
SRC: tyrosine kinase sarcoma
TBA: total bile acid
TBIL: total bilirubin
TEM: transmission electron microscopy
TUNEL: terminal deoxynucleotidyl transferase dUTP nick end labeling
