# Supplementary material for: Low hepatic artery blood flow mediates NET extravasation through the regulation of PIEZO1/SRC signaling to induce biliary complications after liver transplantation
Source: Theranostics. 2024 Oct 14;14(17):6783–97. doi: 10.7150/thno.99514 (PMC11519797; doi:10.7150/thno.99514)
Supplement: Supplementary file 1 — Supplementary materials and methods, figures. [file thnov14p6783s1.pdf]

Supplementary Materials

Low hepatic artery blood flow mediates NET extravasation through the regulation of PIEZO1/SRC signaling to induce biliary complications after liver transplantation

Hongqiang Yu[1, #], Yujun Zhang[1, #], Ling Shuai[1], Cong Peng[1], Changchun Zhao[2], Yan Jiang[1], Ling Yao[1], Jiejuan Lai[1], Zhiyu Chen[1], Leida Zhang[1]*, Xiang Xiong[1]*, Xiaojun Wang[1]*

[1]Key Laboratory of Hepatobiliary and Pancreatic Surgery, Institute of Hepatobiliary Surgery, Southwest Hospital, Third Military Medical University (Army Medical University), Chongqing, China 400038, China

[2] Department of General Surgery, Traditional Chinese Medicine Hospital, Chongqing, China 400015, China

[#] These authors contributed equally to this work

***Corresponding author**:

Professor Lei-da Zhang, E-mail: 2518569931@qq.com

Professor Xiang Xiong, E-mail: xiongxiang@tmmu.edu.cn

Professor Xiao-jun Wang, E-mail: xiaojunwang@tmmu.edu.cn

Tel.: +86-23-68765738

Materials and methods

Experimental animals

Male Sprague–Dawley (SD) rats (200–220 g) (SJA Animal Laboratory, Hunan, China) were used for all experiments. The animals were maintained on a standard laboratory rodent chow diet and in a specific feeding environment. All experiments were approved by the animal ethics committee (AMUWEC20232992).

Rat orthotopic liver transplantation (OLT) model

The rats were anesthetized with isoflurane, their hair was removed, they were immobilized and disinfected, and laparotomy was performed for subsequent surgical operations. The anesthesia dosage was adjusted on the basis of changes in the animal respiratory rate and liver color. After laparotomy, the donor animals received 2 ml of heparinized saline (125 U/ml) via the inferior vena cava (IVC). Cardiac arrest was induced by isoflurane after the injection of heparin for 5 min. The period of in situ warm ischemia started at cardiac arrest, and no surgical operation occurred before or during warm ischemia. Livers were covered during the ischemic period, and the liver temperature was monitored (28 ± 2 °C). After 30 min of in situ ischemia, standard OLT in SD rats was performed according to the cuff technique described by Kamada [1]. The surgical procedure comprises three stages: donor surgery, donor preparation and recipient surgery.

After ischemia, the livers were flushed with cold 0.9% sodium chloride solution at room temperature via the abdominal aorta. Then, the liver was mobilized by sectioning its ligaments and ligating the left diaphragmatic vein, right kidney and adrenal veins. After perfusion, the intrahepatic and suprahepatic vena cava, portal vein, hepatic artery and bile duct were carefully disconnected and explanted into a 5 cm sterile Petri dish with cold 0.9% saline solution, and the preparation of the donor liver was completed. The intrahepatic vena cava and portal vein were cannulated with high-density polyethylene (HDPE) sleeves. The length and inner and outer diameters of the sleeves connected to the infrarenal vena cava were 3 mm, 2.3 mm and 2.8 mm, and those of the sleeves connected to the portal vein were 3 mm, 2 mm and 2.3 mm. Sleeves of the intrahepatic vena cava and portal vein were installed via nondestructive bulldog pliers that are used for stable handling of the sleeve. The blood vessel wall was lifted through the inner cavity of the sleeve via two micro surgical forceps, turned outward to cover the sleeve, and fixed with 8–0 silk thread around the scratched sleeve (Figure S1A). The bile duct was connected with a polyethylene (PE50, USA) catheter (lengths of 3–5 mm, inner and outer are 0.58 mm and 0.96 mm, respectively). The hepatic artery was connected with a polyvinyl chloride (UT-03, Japan) tube (lengths of 3–5 mm; inner and outer lengths of 0.18 mm and 0.3 mm, respectively). For installation of the hepatic bile duct and hepatic artery, a half catheter was directly implanted into the bile duct or hepatic artery via the above wedge-shaped incision and then ligated and fixed with 8–0 silk thread.

After the sleeves of the intrahepatic vena cava and portal vein were installed and the bile duct and hepatic artery were cannulated, the liver was then stored in 0.9% saline solution for 15 ± 2 min until implantation in the recipient. For the recipient liver, a midline longitudinal incision was made in the abdomen of the SD rats, and the abdominal wall was pulled apart to both sides via a custom-made hook. The xiphoid was clamped with curved hemostatic forceps and turned toward the head. The intestinal tract was covered with wet saline gauze and slightly pushed toward the lower left abdomen. The hepatic artery and extrahepatic bile duct at the entrance to the liver were ligated with 8–0 silk thread and cut. The anhepatic phase was initiated after the IVC and portal vein of the recipient blocked with a hemostatic clip, and, a 0.9% sodium chloride solution was injected through

the bifurcation of the left and right lobes of the portal vein to drive out the blood accumulated in the rat vasculature. The suprahepatic vena cava was blocked with vena cava forceps. Hepatectomy was completed by sectioning the hepatic artery, bile duct, portal vein and infrarenal and suprahepatic vena cava, and the donor liver was implanted. The suprahepatic vena cava of the graft in the recipient was continuously anastomosed via 8–0 suture threads under a microscope. Moreover, the suprahepatic vena cava was filled with 5 U/ml heparin saline to prevent blood clots and bubbles. After the portal vein and infrahepatic vena cava were bleed appropriately and the lumen was rinsed with heparin water, the donor sleeve handle was clamped with small bulldog forceps, the vascular wall of the recipient was lifted with micro surgical forceps, the sleeve was placed into the vascular lumen, and the portal vein and infrahepatic vena cava were fixed with 8–0 silk thread. The blocking clips of the portal vein and hepatic IVC were released to end the anhepatic phase. Finally, the catheter at the other end of the donor's hepatic artery and bile duct was connected to the recipient following the above steps for implanting the catheter into the donor (Figure S1A). The period of transplantation was 12–15 min from the blocking IVC to the connecting bile duct. The experimental set up included the following groups (Figure S1B).

- For the warm ischemia-free liver transplantation (WFLT) group with 0 min of DCD donor situ warm ischemia and 15 ± 2 min of cold storage (WFLT; n = 6).

- For the warm ischemia liver transplantation (WLT) group with 30 min of DCD donor situ warm ischemia and 15 ± 2 min of cold storage (WLT; n = 9).

- For the warm ischemia liver transplantation and verapamil treatment group, we used 30 min of DCD donor situ warm ischemia and 15 ± 2 min of cold storage, and the receptor was pretreated with 1.5 mg/kg verapamil hydrochloride (MCE, USA) dissolved in water by gavage for 1 h before surgery (verapamil; n = 9).

- For the warm ischemia liver transplantation and sivelestat treatment group with 30 min of DCD donor situ warm ischemia and 15 ± 2 min of cold storage, and the receptor was treated with 50 mg/kg sivelestat (MCE, USA) by intraperitoneal injection [2] after liver transplantation (sivelestat; n = 8).

Transplantation injury, biliary injury, liver function, and immune system activation were assessed in all four experimental groups at 6 h after transplantation. For longer observation, the rats were treated with 1.5 mg/kg verapamil daily and sacrificed in the fourth week, and abnormal biliary proliferation and fibrosis of the graft liver were analyzed.

Evaluation of hepatic artery blood flow via laser speckle imaging

The rat hepatic artery and intrahepatic blood flow were recorded at various time points before and after liver transplantation and sham operations via an RFLSI ZW laser speckle system and review software (RWD, Shenzhen, China). The rats were anesthetized with isoflurane and immobilized on a warming plate maintained at 37 °C. For hepatic artery blood flow measurements, Custom-made hook were used to retract the abdominal wall to both sides to fully expose the liver and hepatic artery. Data were acquired from a 20 mm × 15 mm field of view via a 785 nm, 90 mW laser with a sampling rate of 0.1 Hz for 5 min at a working distance of 260 mm. Flux over time was analyzed by LSCI review software. In each visible hepatic artery and surface of liver, regions of interest (ROI) were measured based on the intensification of blood flow. All assessments and analyses were performed in a blinded manner.

Biochemical parameter assay and ELISA assay

Biochemical parameters of hepatobiliary function were evaluated using a Pre-Analytical Process Automation System in the Clinical Laboratory Department of Southwest Hospital. Levels of alanine aminotransferase (ALT), aspartate aminotransferase (AST), gamma glutamyltranspeptidase (GGT), direct bilirubin (DBIL), total bilirubin (TBIL), total bile acid (TBA), alkaline phosphatase (ALP) and lactate dehydrogenase (LDH) were measured at 0.25, 1, 2, 3, 4, 5, 6, 7 and 28 days after liver transplantation. The plasma samples from grafted rats were collected by centrifugation from EDTA anticoagulated blood and the NET components and level were detected via ELISA. A rat myeloperoxidase ELISA kit (Abcam, USA) and Quant-iTTM PicoGreen double-stranded DNA (dsDNA) detection kit (Thermo Fisher Scientific, USA) were used to assess and quantify MPO and dsDNA. A rat C3a and C5a ELISA kits (FineTest, Wuhan, China) were used to assess and quantify C3a and C5a following the instructions of the manufacturers.

Proteomic analysis

The proteomic sequencing of the liver at 6 h post-surgery was completed via the BGI platform (Beijing Genomics institution, China). Peptide detection and proteomic analyses were described by previous researchers [3-5]. Briefly, approximately 2 mg (in some cases <2 mg) of snap-frozen liver biopsies were cryopulverized and transferred to an Eppendorf tube. The cell samples were sonicated 3 times on ice via a high-intensity ultrasonic processor in lysis buffer (4% SDS and 1% protease inhibitor cocktail). The remaining debris was removed by centrifugation at 25,000 × g at 4 °C for 15 min. Then, the supernatants were collected and digested by incubation with 10 mM dithiothreitol at 37 °C for 30 min, followed by alkylation with 55 mM iodoacetamide at room temperature in the dark for 45 min. After incubation with precooled acetone at -20 °C for 2 h, the supernatants were removed by centrifugation at 25,000 × g at 4 °C for 15 min. Finally, the concentration of the protein lysate was measured via a Bradford assay according to the manufacturer's instructions. For proteolysis, a protein mixture containing 0.25% trypsin (Gibco, USA) was incubated at 37 °C for 4 h, after which the peptide mixtures were collected into new collection tubes by centrifugation at 14,000 × g for 15 min at 25 °C. The peptide mixture was manually fractionated into high-pH reversed-phase mixtures with stepwise gradients. The C18 tip packed with 5 mg of C18 reverse-phase media was washed with methanol and ammonia water. Then, 50 µg of peptide redissolved in ammonia water (pH 10) was added. The tip was subsequently centrifuged at 1,000 × g for 8 min at 25 °C to remove the liquid, followed by washing with ammonia water. The peptides bound to the C18 reverse-phase packing were then sequentially eluted with different concentrations of acetonitrile in ammonia water. These fractions were collected and heat-dried and stored at -80 °C.

For analysis of the peptide mixture of each tissue after liver transplantation, an LC–MS/MS system consisting of a nanoflow high-performance liquid chromatography instrument (EASY-nLC 1000 nanoflow LC, Thermo, USA) was used. Each fraction of the peptide mixture was redissolved in mobile phase A (0.1% formic acid, 99.9% pure water), loaded onto the trapping column via mobile phase A and then separated on an analytical column (75 µm × 250 mm, ReproSil-Pur C18-AQ, 1.8 µm; GmbH, Germany) at a flow rate of 300 nl/min with the following gradient: 0 min, 2% mobile phase B (0.1% FA in 99.9% acetonitrile); 0–45 min, 2–22% mobile phase B; 45–50 min, 22–35% mobile phase B; 50–55 min, 35–80% mobile phase B; and 55–60 min, 80% mobile phase B.

For data-dependent acquisition (DDA) and data-independent acquisition (DIA) analyses, the DDA raw files were searched against the human UniProt database (updated 2022-09-07, containing 20,398 protein groups and the iRT peptide sequence) with MaxQuant version 2.0.3.0. The DIA raw files in the liver biopsy dataset were analyzed with Spectronaut v.13. Data filtering was set to 'Q

value'. 'Cross-run normalization' was enabled with the strategy of 'local normalization' on the basis of rows with 'Q value complete'. The precursor false discovery rate (FDR) was set to 1% at both the protein and peptide precursor levels. A previously generated deep fractionated DDA library and liver DDA library were used in the targeted analysis of DIA data for liver datasets. For preprocessing of liver proteomic datasets, the peptides and proteins were identified and quantified from the DDA spectral library via deconvolution of the DIA data. The MSstats software package was used to perform differential analysis [6]. Proteomic datasets of the liver were filtered for 60% valid values across all samples (proteins with >40% missing values were excluded from downstream statistical analysis), with the remaining missing values imputed by drawing random samples from a normal distribution. Proteins whose fold change value was greater than 1.5 and p value was less than 0.05 were chosen as differentially expressed proteins, followed by functional analysis of these proteins.

Transmission electron microscopy

After liver transplantation for 6 h, 1 mm thick slices of rat liver tissue samples were prepared for transmission electron microscopy (TEM). The tissue samples were fixed (2.5% glutaraldehyde, 2% paraformaldehyde, 0.1 M sodium cacodylate buffer, pH 7.4) immediately after euthanasia and then dissected overnight at 4 °C. After subsequent buffer washes, the samples were post-fixed in 2.0% osmium tetroxide for 1 h at room temperature and rinsed with 2% uranyl acetate. After dehydration through a graded ethanol series, the tissue was infiltrated and embedded. ultrathin sections were prepared via a microtome (Leica EM UC7 RT, Leica, Vienna, Austria), stained with uranyl acetate and lead citrate, and scanned under an electron microscope (JEM 1400PLUS, Jeol, Akishima, Japan) operating at 100 kV, and photographed. Abundant scattered apical microvilli (MV) and cell junction indicated normal physiologic function of bile duct [7].

Histological and immunohistochemical staining analysis

Rat liver tissue samples after liver transplantation were fixed in 10% formalin for 1 week, dehydrated for 11 h, and embedded in paraffin (Leica, USA). Paraffin-embedded rat tissue samples were cut into 3 μm thick tissue sections, mechanically deparaffinized, and stained with hematoxylin and eosin (H&E) for histological analysis according to standard protocols. For immunohistochemical staining, the tissue sections were incubated in 2% sodium citrate antigen retrieval solution (Solarbio, USA) at high temperature and pressure for 2.5 min; then, the samples were washed 3 times for 5 min each with phosphate-buffered saline (PBS). Endogenous peroxidase activity was blocked with methanol containing 5% hydrogen peroxide for 10 min, and the samples were washed 3 times for 5 min each with PBS and incubated with a solution containing 5% bovine serum albumin (Biofroxx, Germany) for 30 min at room temperature. Then, the tissue sections were incubated with primary antibodies at 4°C overnight, followed by incubation with a secondary antibody at room temperature for 30 min. Next, the sections were stained with DAB (ZSGB-BIO, Beijing, China). The sections were subsequently counterstained with hematoxylin (Biosharp, Beijing, China) for 30 seconds and incubated in warm water for 20 min. The tissue sections were dehydrated and preserved in neutral balsam (Biosharp, Beijing, China) at room temperature. Rabbit anti-myeloperoxidase (MPO) (ab208670), rabbit anti-NE (ab310335), rabbit anti-CK19 (ab52625), rabbit anti-CK7 (ab181598) and rabbit anti-CD31 (ab281583) antibodies were purchased from Abcam Company (USA). Rabbit anti-PIEZO3 (15939-1-AP) antibody was obtained from Proteintech Company (Wuhan, China). Rabbit anti-SRC (2109) and rabbit anti-p-SRC (2105) antibodies were purchased from Cell Signaling Technology. The secondary antibody was purchased from Dako. The staining was evaluated by

different specialized pathologists who were blinded to the patient characteristics. The staining intensity and number were determined from 3 different areas for each sample. Ischemia-reperfusion-associated lesions in the liver tissue, such as sinusoidal congestion, cytoplasmic vacuolization, and necrosis of hepatic cells, were scored with the Suzuki 0–4 scale of ascending extent and severity as previously described [8]. Inhepatic and extrahepatic bile duct injuries in biopsies were semiquantified by calculating the bile duct injury severity score (BDISS) and bile duct damage score (BDDS), respectively [9, 10]. Intrahepatic biliary and hepatocytic apoptosis was examined histologically via TUNEL assays of liver sections with a colorimetric TUNEL apoptosis assay kit (Beyotime, Shanghai, China) according to the manufacturer's protocol. A Dako REAL EnVision kit (K5007, Dako, Denmark) was then used for immunohistochemical staining with primary antibodies to determine the protein levels. Four weeks after transplantation, the hepatic tissue sections were stained with Sirius Red solution (Solarbio, USA) for approximately 30 min at room temperature. The degree of hepatic fibrosis in the rats was determined via the METAVIR scoring system [11].

Immunofluorescence assay

For paraffin-embedded rat tissue samples, non-specific protein binding was blocked by 5% bovine serum albumin for 30 min after retrieved antigen and blocked endogenous peroxidase activity. The samples were incubated overnight with primary antibodies. Then, the samples were washed 3 times for 5 min each with PBS and incubated with labeled isotype-specific fluorescent secondary antibodies (AiFang, Hunan, China) for 1 h at room temperature. The samples were incubated with another primary antibody and the above steps were repeated. The primary antibodies used were as follows: rabbit anti-MPO, rabbit anti-CK19, rabbit anti-α-SMA (ab5694, Abcam, USA), rabbit anti-NE (ab314916, Abcam, USA), rabbit anti-F4/80 (ab300421, Abcam, USA), mouse anti-Ly6G (NBP3-27164, Novus, USA), mouse anti-Integrin β2 (sc-8420, Santa Cruz, USA), mouse anti-ICAM-1 (sc-8439, Santa Cruz, USA), mouse anti-C3 (sc-28294, Santa Cruz, USA) and rabbit anti-C5 (ab275931, Abcam, USA). Next, the samples were incubated with Hoechst33342 (Invitrogen, USA) to label cell nuclei for 10 min at room temperature and storeged with antifade mounting medium (Beyotime, Shanghai, China). For all immunoreactions, negative controls were also prepared by replacing the primary antibody with pre-immune serum. The slides were further scanned via a digital scanner (Olympus, Germany) and processed by Image J software. For rat neutrophils, the cells were isolated from peripheral blood post-surgery via neutrophil isolation kit. Then, the cells were counted via a cells counter and smeared onto polylysine-coated glass slides. The cells were subsequently fixed with 4% paraformaldehyde for 40 min after being dried at room temperature. The cells were permeabilized with Triton X-100 for 15 min and blocked for 30 min with 5% bovine serum albumin. The cells were incubated with primary antibodies at 4°C overnight, and secondary antibodies conjugated to fluorophores were incubated at room temperature for 1 h. Finally, the nuclei of the cells were stained with Hoechst33342 or Sytox Green (Invitrogen, USA). Images of the cells were captured via Olympus microscope or Zeiss confocal microscope, to assessed the level of formed NET isolated from peripheral blood.

Neutrophil isolation and Giemsa staining

The neutrophil isolation procedure was as described previously. Briefly, cells were separated into single-cells from rat peripheral blood after liver transplantation via neutrophil isolation kit (TBD, Tianjin, China). Then, the cells were counted by a cell counter and frozen in liquid nitrogen

with cell preservation solution. For Giemsa staining, neutrophil cells were smeared onto polylysine-coated glass slides, fixed with methanol and then incubated with a Giemsa stock solution (Solarbio, USA) following the manufacturer's instructions. The images were visualized under an Olympus microscope and photographed with a digital camera. Abnormal neutrophils were characterized by a lack of nuclear segmentation and an increase of extracellular cytoplasmic extensions [12].

Primary liver sinusoidal endothelial cell isolation

The isolated steps of primary rat liver sinusoidal endothelial cell (LSEC) involve in circulating perfusion and tissue separation in vivo [13] and gradient centrifugation via density percoll in vitro [14] after liver transplantation. The rats were anesthetized with isoflurane, disinfected, immobilized, and subjected to laparotomy to expose the inferior vena cava. A 22-gauge catheter was cannulated into the IVC and ligated with surgical thread to prevent dislocation. The liver was disconnected and explanted into a sterile Petri dish. The catheter was connected to a perfusion setup. An EGTA solution, pronase solution (0.4 mg/ml) and collagenase solution (0.8 mg/ml) filtered through a 0.2 μm filter were circulating perfused for 2 min, 5 min and 7 min in three dish, respectively. After perfusion in situ, the catheter was removed from the IVC. The livers were transferred into a dish containing 5 ml of pronase/collagenase solution (containing 4 mg of pronase, 5 mg of collagenase and 1% DNase I) and cut into pieces for digestion in vitro for 10 min. The digested rat livers were filtered through a 70 μm cell strainer into a 50 ml tube. Liver cell suspension was centrifuged for 5 min at 50 × g (4 °C) to separate hepatocytes and non-parenchymal cells (NPC). The NPC supernatant was transferred into 50 mL conical tubes and centrifuged at 300 × g for 10 min. The supernatant was discarded and the pellet was resuspended in 15 ml ice cold PBS. A percoll gradient was prepared in 50 ml conical tubes with 15 ml of 50% percoll (isotonic) at the bottom and 20 ml of 25% percoll (isotonic) layered on top. 15 ml of the NPC fraction (in PBS) was carefully placed on the 25% percoll layer and centrifuged at 900 × g for 25 min (without brake). At the end of the centrifugation, the layer between the 25%–50% percoll gradient (10–15 ml) was collected, diluted in ice cold PBS and centrifuged at 900 × g for 10 min (4 °C) to pellet the cells. Any remaining percoll was aspirated and the cells were suspended in fresh hepatocyte culture media (10 ml). To remove any contaminating cells (Kupffer), the cell fraction was incubated on a 10 cm diameter tissue culture dish for 15 min (37 °C) and the non-adherent cells (LSEC) were collected and immediately utilized for preparing the western blotting assay.

Flow cytometric analysis

For flow cytometric analysis to assessed the NET level in peripheral blood after liver transplantation, neutrophil cells were isolated via a neutrophil isolation kit. Subsequently, the cells were fixed with 4% paraformaldehyde for 15 min and permeabilized with Triton X-100 for 15 min at room temperature. Single-cell suspensions were washed 3 times for 5 min each with PBS, and then surface staining was performed with mouse anti-Ly6G antibodies (NBP3-27164, Novus, USA) for 1.5 h at 37 °C. Next, the cells were incubated with primary antibodies, namely, rabbit anti-MPO (1:400) for 1.5 h at 37°C, and then incubated with secondary antibodies conjugated to fluorophores at room temperature for 30 min. The stained single-cell suspensions were analyzed using a BDFAcsAriaII flow cytometer (BD Biosciences, CA, USA), and the results were analyzed with FlowJo 10.0 software (FlowJo, LLC, OR USA).

Cell culture studies and apoptosis, MTT and NET formation assay

The human intrahepatic cholangiocarcinoma cell line HuCCT1 was obtained from the Bohu Biotechnology Company (Shanghai, China). All cell lines tested were negative for mycoplasma contamination. All the experiments were conducted in Dulbecco's Modified Eagle's Medium (DMEM) supplemented with 10% fetal bovine serum (Gibco, New Zealand), 100 U/ml penicillin, and 100 mg/ml streptomycin (Invitrogen, USA) at 37 °C, 95% humidity, and 5% CO_2. For the apoptosis experiments, HuCCT1 cells (1×10^5 cells per well) were co-cultured for 24 h with activated neutrophil (3×10^5) isolated from rat peripheral blood after liver transplantation in a 12-well plate. Apoptosis was measured via annexin V-FITC apoptosis detection kit (Beyotime, Shanghai, China), which was employed to assess the levels of detectable phosphatidylserine externalization on the outer membrane of apoptotic cells. After removing the culture medium, the cells were gently washed once with PBS, gently suspended in 1 × binding buffer (500 μl) and incubated with 5 μl of annexin V-FITC and 5 μl of PI (50 μg/ml) in dark for 15 min following the step by step protocol provided by the manufacture. Next, the cells were subsequently darkly fixed with 4% paraformaldehyde for 40 min at room temperature. Thereafter, the extent of apoptosis was subjected to an immediate analysis with Olympus fluorescent microscope. The cell viability was evaluated using the standard colorimetric MTT assay according to the manufacturer's protocol, which is based on the conversion of yellow MTT to purple formazan derivatives by mitochondrial succinate dehydrogenase in viable cells. Briefly, HuCCT1 cells (3×10^3 cells per well) were co-cultured with activated neutrophil (6×10^3) for 24 h in a 96-well plate. Afterwards, cells were treated with the addition of 25 μl MTT solution (5 mg/ml) to each well in the dark at 37 °C for another 4 h incubation. At the end of experiment, the plates were centrifuged to discard the MTT. Subsequently, 200 μl stop solution DMSO was added to dissolve the formazan crystals. The absorbance was measured at 570 nm via an ELISA reader (Thermo Fisher Scientific, USA). The percent viability of the treated cells was defined as the ratio of the absorbance of treated cells to that of untreated control cells. For the NET formation experiments, Neutrophils isolated from the peripheral blood of health rat were seeded (6×10^5 cells/ml) into 6 well plates with or without cell slides in RPMI medium supplemented with 2 % fetal bovine serum, and 1 % penicillin–streptomycin. The cells were allowed to adhere for 30 min and then stimulated with 100 nM C3a or C5a (FineTest, Wuhan, China) in a 5% carbon dioxide atmosphere at 37 °C for 6 h [15, 16]. Next, the cells were subsequently darkly fixed with 4% paraformaldehyde for 40 min and incubated with 5 μM SYTOX™ Green nucleic acid stain (Invitrogen, USA) for 60 min at room temperature. Formatted NETs was visualized with a fluorescent microscope, and the NET percentage was calculated by semi-quantitative analysis of the SYTOX™ positive NETs (Filamentous structure) in 6 non-overlapping fields. Meanwhile, the stimulated cells were collected and used immediately via western blotting assay [12, 17].

Western blotting analysis

Treated rat liver tissue samples, isolated liver sinusoidal endothelial cells and isolated peripheral neutrophil cells after liver transplantation were lysed with RIPA lysis buffer (Thermo Fisher Scientific, USA) supplemented with a protease and phosphatase inhibitor mixture (Roche, Switzerland) and then incubated at 4 °C for 30 min. After incubation, the lysates were centrifuged at 13,000 rpm for 10 min at 4 °C. After centrifugation, the supernatants were transferred to new Eppendorf tubes. The protein concentrations were quantified via a BCA protein assay reagent kit (Beyotime, Shanghai, China). Denatured samples were separated by SDS–PAGE (Beyotime, China) and then transferred to NC membranes (GE Healthcare, UK). The NC membranes were incubated

with the following antibodies for western blotting at 4 °C overnight: goat anti-rabbit IgG HRP (7074), rabbit anti-SRC antibody (2109) and anti-p-SRC antibody (2105) antibodies were purchased from Cell Signaling Technology Company (Danvers, MA); rabbit anti-PIEZO1 antibody (15939-1-AP), rabbit anti-CD31 antibody (28083-1-AP) and rabbit anti-GAPDH antibody (10494-1-AP) were obtained from Proteintech Company (Wuhan, China); mouse anti-C3 (sc-28294), rabbit anti-C5 (ab275931, Abcam, USA) and mouse anti-NE (sc-55549) were obtained from Santa Cruz Company (USA). The NC membranes were incubated with secondary antibodies were conjugated to HRP at room temperature for 1 h. Finally, images of the protein bands were captured via a chemiluminescence imaging system.

Statistical analysis

R (R 4.1.2) and R-Studio (v7.2 Build 153957) software were used to analyze the data and create statistical charts, and the data are presented as the means ± standard errors. For data processing, the R package MSstats (v4.1.1) was used for data normalization and analysis of differentially expressed proteins. Kyoto Encyclopedia of Genes and Genomes (KEGG) and Gene Ontology (GO) analyses were implemented via the R package clusterProfiler (v3.18.1) with the Enricher function. Gene set enrichment analysis (GSEA) was performed via the R package GSVA (v3.18.2) with the GSVA function and GSEA method. Proteins with a fold change value greater than 1.5 and a p value less than 0.05 were defined as differentially expressed proteins. All the analyses were performed with R software. Comparisons between 2 groups were performed with a 2-tailed unpaired t test. The correlations between the number of MPO-positive cells and biliary injury scores were determined via Pearson's correlation coefficient. Overall survival of recipient post-surgery was analyzed via the log-rank test and the Kaplan–Meier method. $p \leq 0.05$ was defined as statistically significant. Area determination was performed via an imaging system (Olympus, Germany) and FIJI software (ImageJ, National Institutes of Health). Adobe illustrator software (Adobe, USA) was used.

Other materials and methods are described in the supplemental materials and methods.

References

1. Kamada N, Calne RY. Orthotopic liver transplantation in the rat. Technique using cuff for portal vein anastomosis and biliary drainage. Transplantation. 1979; 28: 47-50.
2. Okeke EB, Louttit C, Fry C, Najafabadi AH, Han K, Nemzek J, et al. Inhibition of neutrophil elastase prevents neutrophil extracellular trap formation and rescues mice from endotoxic shock. Biomaterials. 2020; 238: 119836.
3. Baker CP, Phair IR, Brenes AJ, Atrih A, Ryan DG, Bruderer R, et al. DIA label-free proteomic analysis of murine bone-marrow-derived macrophages. STAR protocols. 2022; 3: 101725.
4. Niu LL, Thiele M, Geyer PE, Rasmussen DN, Webel HE, Santos A, et al. Noninvasive proteomic biomarkers for alcohol-related liver disease. Nat Med. 2022; 28: 1277-87.
5. Wang MC, Weng S, Li CY, Jiang Y, Qian XH, Xu P, et al. Proteomic overview of hepatocellular carcinoma cell lines and generation of the spectral library. Sci Data. 2022; 9.
6. Choi M, Chang CY, Clough T, Broudy D, Killeen T, MacLean B, et al. MSstats: an R package for statistical analysis of quantitative mass spectrometry-based proteomic experiments. Bioinformatics. 2014; 30: 2524-6.
7. Khandekar G, Llewellyn J, Kriegermeier A, Waisbourd-Zinman O, Johnson N, Du Y, et al. Coordinated development of the mouse extrahepatic bile duct: Implications for neonatal susceptibility to biliary injury. J Hepatol. 2020; 72: 135-45.

8. Suzuki S, Toledo-Pereyra LH, Rodriguez FJ, Cejalvo D. Neutrophil infiltration as an important factor in liver ischemia and reperfusion injury. Modulating effects of FK506 and cyclosporine. Transplantation. 1993; 55: 1265-72.
9. Geuken E, Visser D, Kuipers F, Blokzijl H, Leuvenink HG, de Jong KP, et al. Rapid increase of bile salt secretion is associated with bile duct injury after human liver transplantation. J Hepatol. 2004; 41: 1017-25.
10. Brunner SM, Junger H, Ruemmele P, Schnitzbauer AA, Doenecke A, Kirchner GI, et al. Bile duct damage after cold storage of deceased donor livers predicts biliary complications after liver transplantation. J Hepatol. 2013; 58: 1133-9.
11. Bedossa P, Dargere D, Paradis V. Sampling variability of liver fibrosis in chronic hepatitis C. Hepatology. 2003; 38: 1449-57.
12. Zhang RZ, Su L, Fu M, Wang Z, Tan LD, Chen HJ, et al. CD177 cells produce neutrophil extracellular traps that promote biliary atresia. J Hepatol. 2022; 77: 1299-310.
13. Mederacke I, Dapito DH, Affo S, Uchinami H, Schwabe RF. High-yield and high-purity isolation of hepatic stellate cells from normal and fibrotic mouse livers. Nature protocols. 2015; 10: 305-15.
14. Bale SS, Geerts S, Jindal R, Yarmush ML. Isolation and co-culture of rat parenchymal and non-parenchymal liver cells to evaluate cellular interactions and response. Scientific reports. 2016; 6: 25329.
15. Wu XT, You DY, Cui J, Yang LY, Lin LY, Chen Y, et al. Reduced Neutrophil Extracellular Trap Formation During Ischemia Reperfusion Injury in C3 KO Mice: C3 Requirement for NETs Release. Front Immunol. 2022; 13: 781273.
16. van der Velden S, van Osch TLJ, Seghier A, Bentlage AEH, Mok JY, Geerdes DM, et al. Complement activation drives antibody-mediated transfusion-related acute lung injury via macrophage trafficking and formation of NETs. Blood. 2024; 143: 79-91.
17. Domingo-Gonzalez R, Martinez-Colon GJ, Smith AJ, Smith CK, Ballinger MN, Xia M, et al. Inhibition of Neutrophil Extracellular Trap Formation after Stem Cell Transplant by Prostaglandin E2. American journal of respiratory and critical care medicine. 2016; 193: 186-97.

Supplementary figure and figure legend

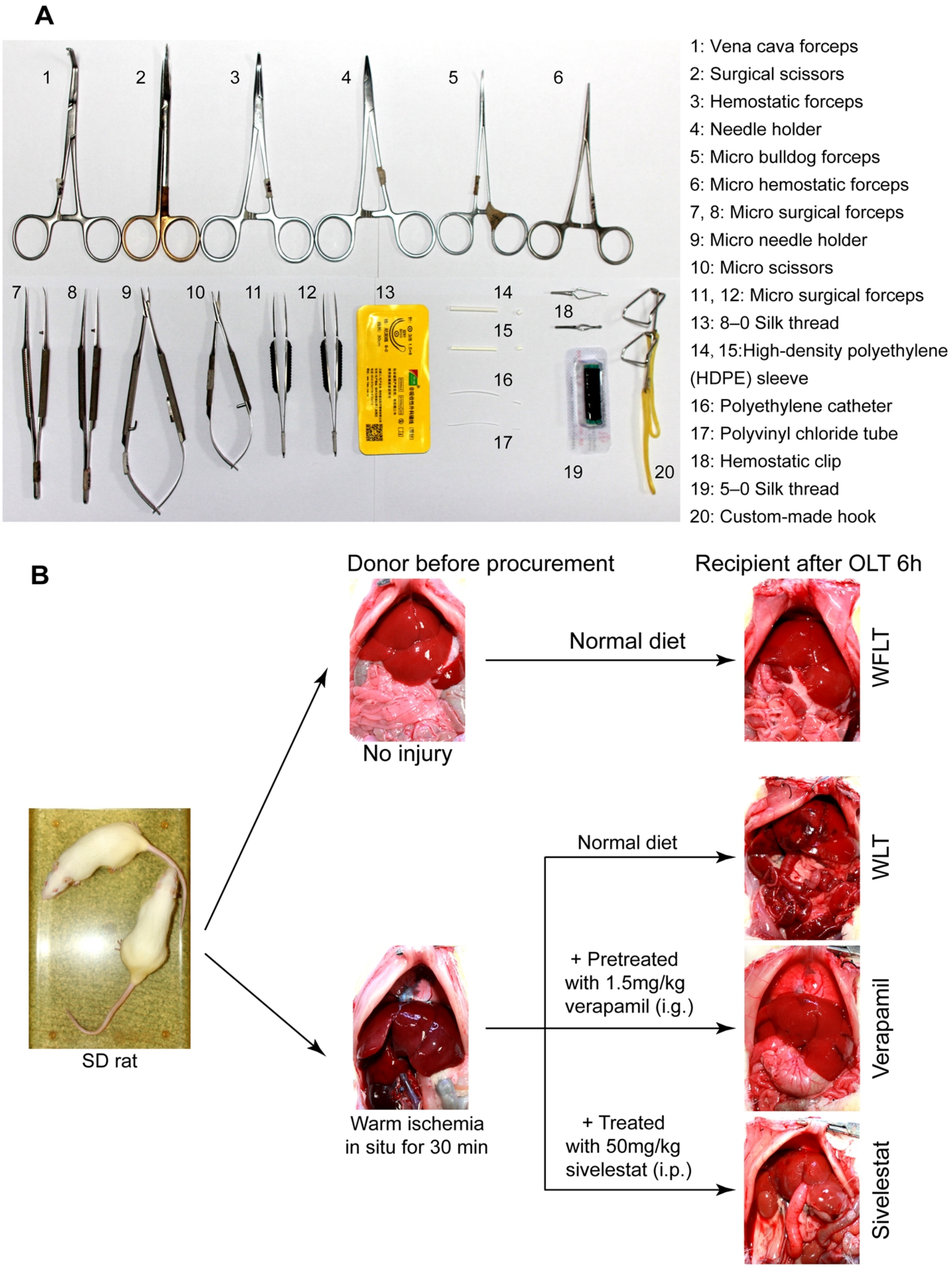

Figure S1. Experimental design. **(A)** Main surgical instruments for rat liver transplantation. **(B)** The male

Sprague-Dawley rats were maintained on a standard laboratory rodent chow diet and in a specific feeding environment. The following experimental groups were included into our experiments:

1) For the warm ischemia-free liver transplantation (WFLT) group with 0 min of DCD donor situ warm ischemia and 15 ± 2 min of cold storage (WFLT; n = 6).

2) For the warm ischemia liver transplantation (WLT) group with 30 min of DCD donor situ warm ischemia and 15 ± 2 min of cold storage (WLT; n = 9).

3) For the warm ischemia liver transplantation and verapamil treatment group, we used 30 min of DCD donor situ warm ischemia and 15 ± 2 min of cold storage, and the receptor was pretreated with 1.5 mg/kg verapamil hydrochloride (MCE, USA) dissolved in water by gavage for 1 h before surgery (verapamil; n = 9).

4) For the warm ischemia liver transplantation and sivelestat treatment group with 30 min of DCD donor situ warm ischemia and 15 ± 2 min of cold storage, and the receptor was treated with 50 mg/kg sivelestat (MCE, USA) by intraperitoneal injection after liver transplantation (sivelestat; n = 8).

The anatomy image of donor and receptor with liver transplantation for 6 h.

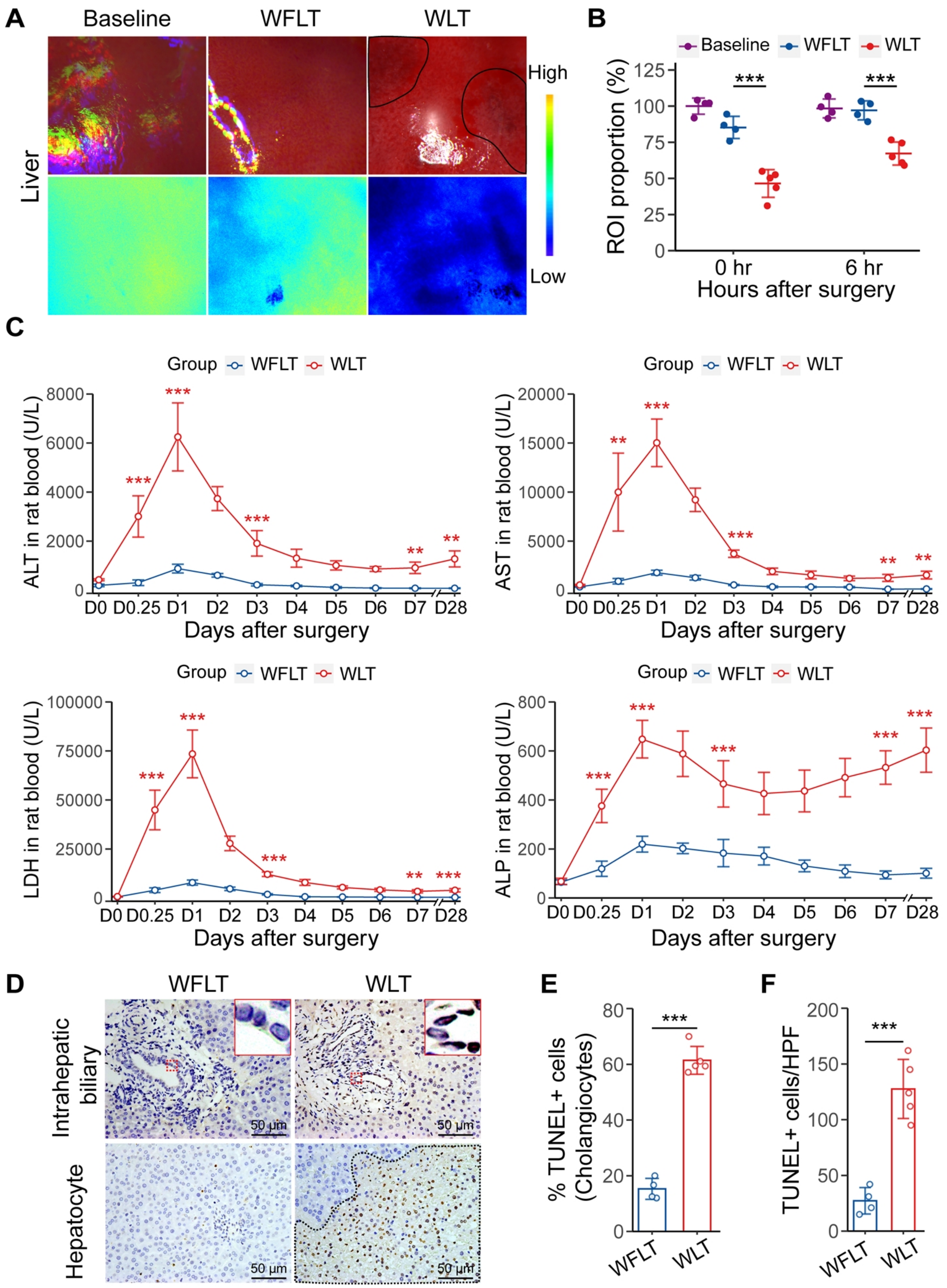

Figure S2. Intrahepatic blood flow and hepatobiliary injury in grafted rats. (A) Representative laser speckle

images of rat hepatic blood flow at 0 h post-surgery. The black outline indicates injured plaques. **(B)** ROI in image A showing the proportions of hepatic blood flow in the baseline group (n = 4), WFLT group (n = 4) and WLT group (n = 5). **(C)** Clinical parameter measurements of hepatobiliary function (n = 4-6 rat per time point) in rat peripheral blood after liver transplantation. **(D)** The TUNEL image of liver tissues from intrahepatic biliary cells and hepatocytes. The red dashed outline and black dashed outline signed positive biliary cells and positive hepatocyte, respectively. Scale bar: 50 µm. **(E)** Quantification of the percent of TUNEL-positive biliary cells in image D. **(F)** Quantification of TUNEL-positive hepatic cells of high powered field (HPF) in image D. Mean values ± SD. **p < 0.01; ***p < 0.001.

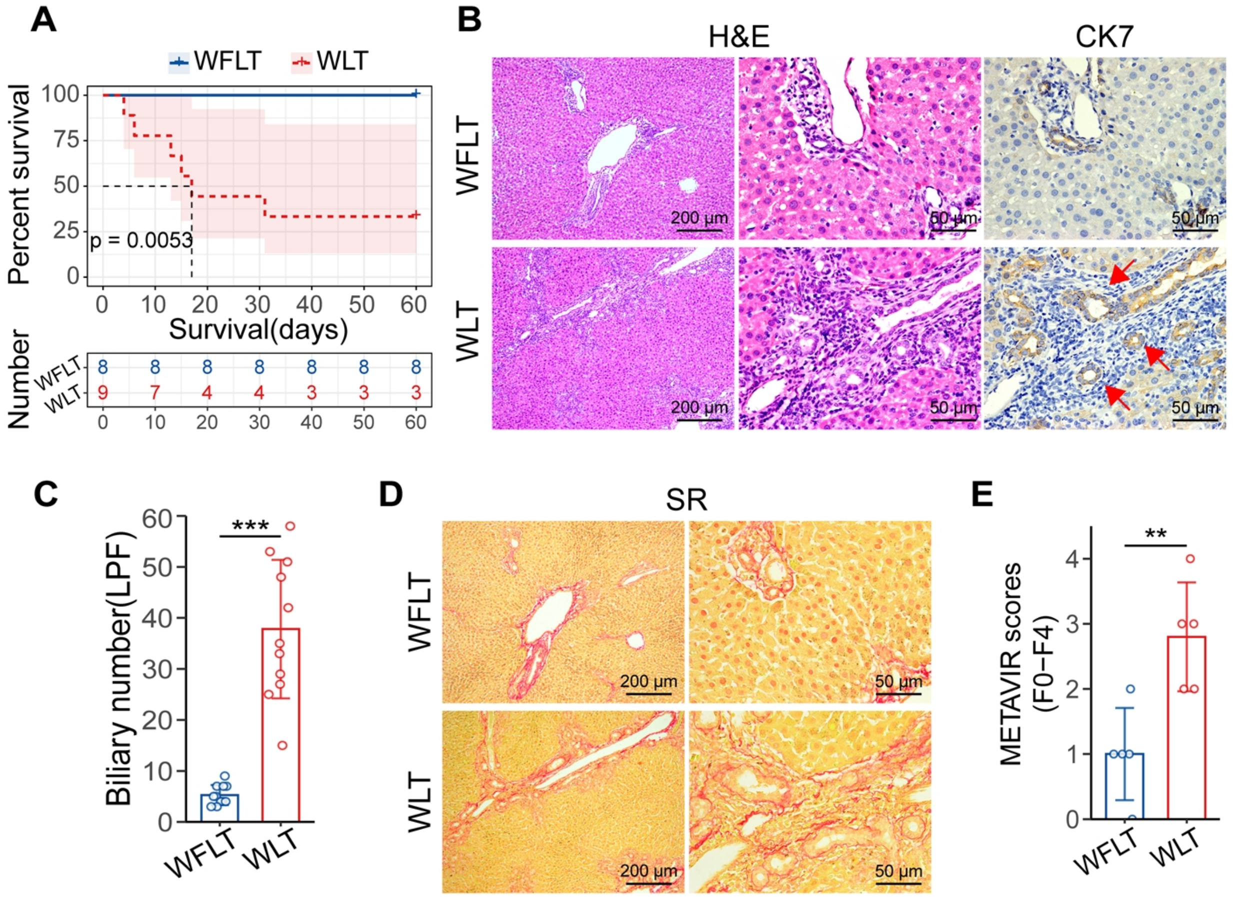

Figure S3. Biliary complications after liver transplantation. (A) Kaplan–Meier plot depicting the survival pattern of recipients in WFLT group (n = 8) and WLT group (n = 9). **(B)** Tissue section images of H&E and immunohistochemistry staining image of grafted liver tissue from the bile duct (indicated by CK7) after four weeks. **(C)** Quantification of CK7-positive cells of low powered field (LPF) in image B. **(D)** Sirius Red (SR) staining images of grafted liver tissue after surgery. **(E)** Fibrosis scores of Sirius Red-positive areas in image D via METAVIR score. Scale bar: 200 µm and 50 µm. Mean values ± SD. **p < 0.01; ***p < 0.001.

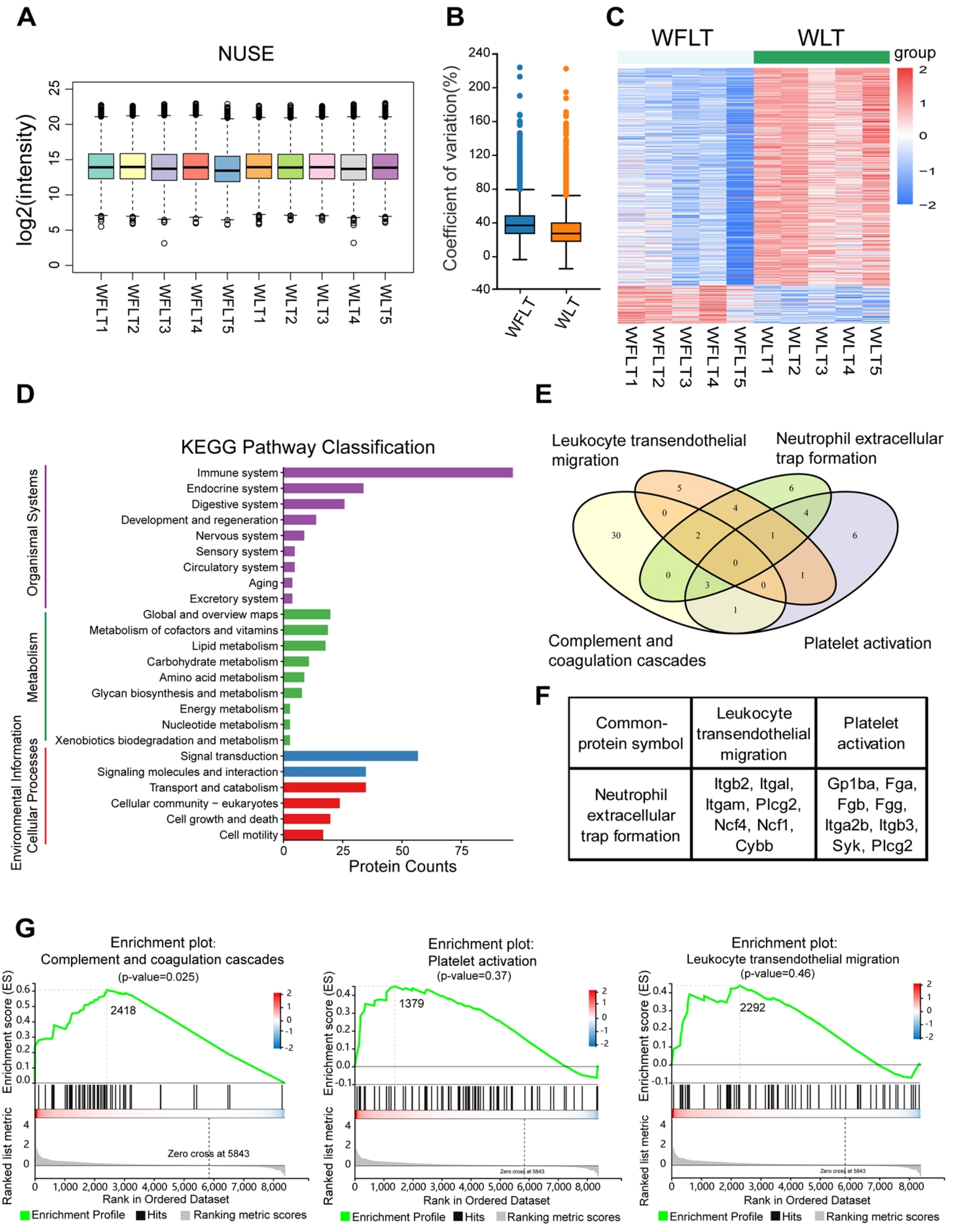

Figure S4. Proteomic map analysis in rat liver transplanted model. (A) Normalized unscaled standard errors (NUSE) boxplot of ten samples. **(B)** Coefficient of variation (CV) values boxplot in the WFLT and WLT group. **(C)** Heatmap of differential proteins (p value < 0.05) between the WFLT and WLT group. The color scale bar indicates the range of proteins (red indicates high expression and blue indicates low expression). **(D)** KEGG

enrichment barplot of differentially expressed proteins (absolute log2-fold change value > 2, p value < 0.05) in liver tissue. **(E)** Venn diagram indicating the number and **(F)** protein symbols of common upregulated proteins in NET formation, platelet activation, and transendothelial migration pathway. **(G)** GSEA enrichment image of complement and coagulation cascades, platelet activation, and transendothelial migration pathways.

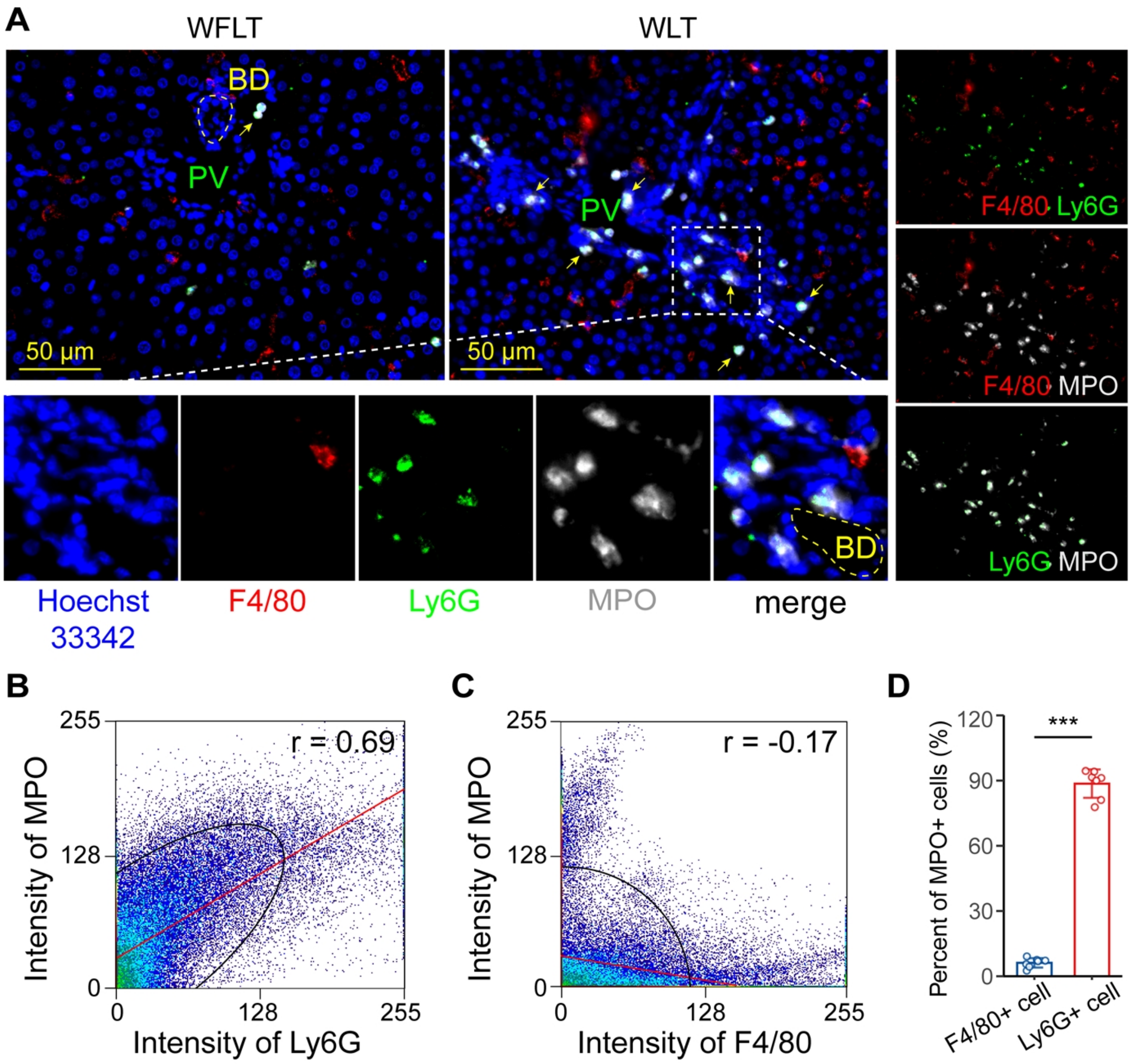

Figure S5. **(A)** Immunofluorescence staining image of liver tissue samples from intrahepatic neutrophil (indicated by LY6G), macrophage (indicated by F4/80) and NETs (indicated by MPO) at 6 h post-surgery. Blue indicate the cellular nucleus, and yellow arrows indicate the co-expressed area. Scale bar: 50 μm. **(B)** Immunofluorescence relationship image of MPO with Ly6G, and **(C)** with F4/80 in image A. The black curve indicates the 90% confidence interval, and the red diagonal line indicates the correlation coefficient. **(D)** Quantitative analysis of the co-localized signal in image A. BD, bile duct; PV, portal vein. Mean values ± SD. ***p < 0.001.

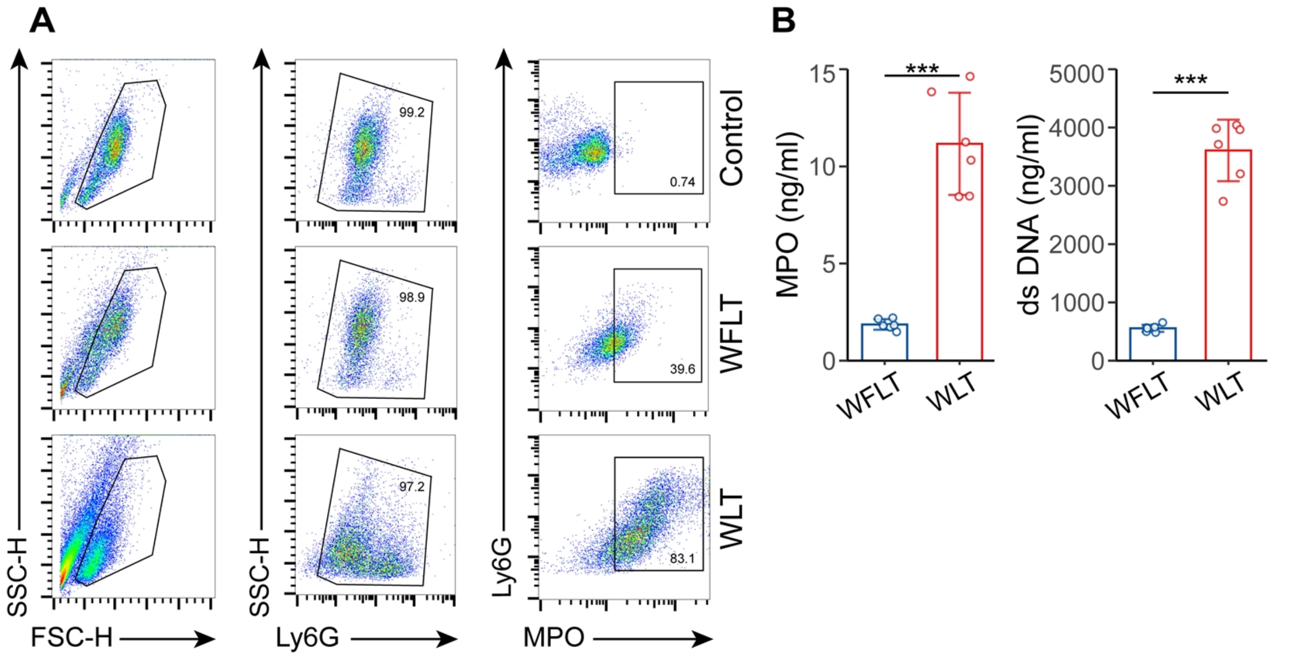

Figure S6. **(A)** Flow cytometric analysis of Ly6G+ and MPO+ cells in the peripheral blood of post-surgery rats. **(B)** Levels of MPO and dsDNA in the rat peripheral blood were detected via ELISA and dsDNA detection kit (n = 6) at 6 h post-surgery, respectively. Mean values ± SD. ***$p < 0.001$.

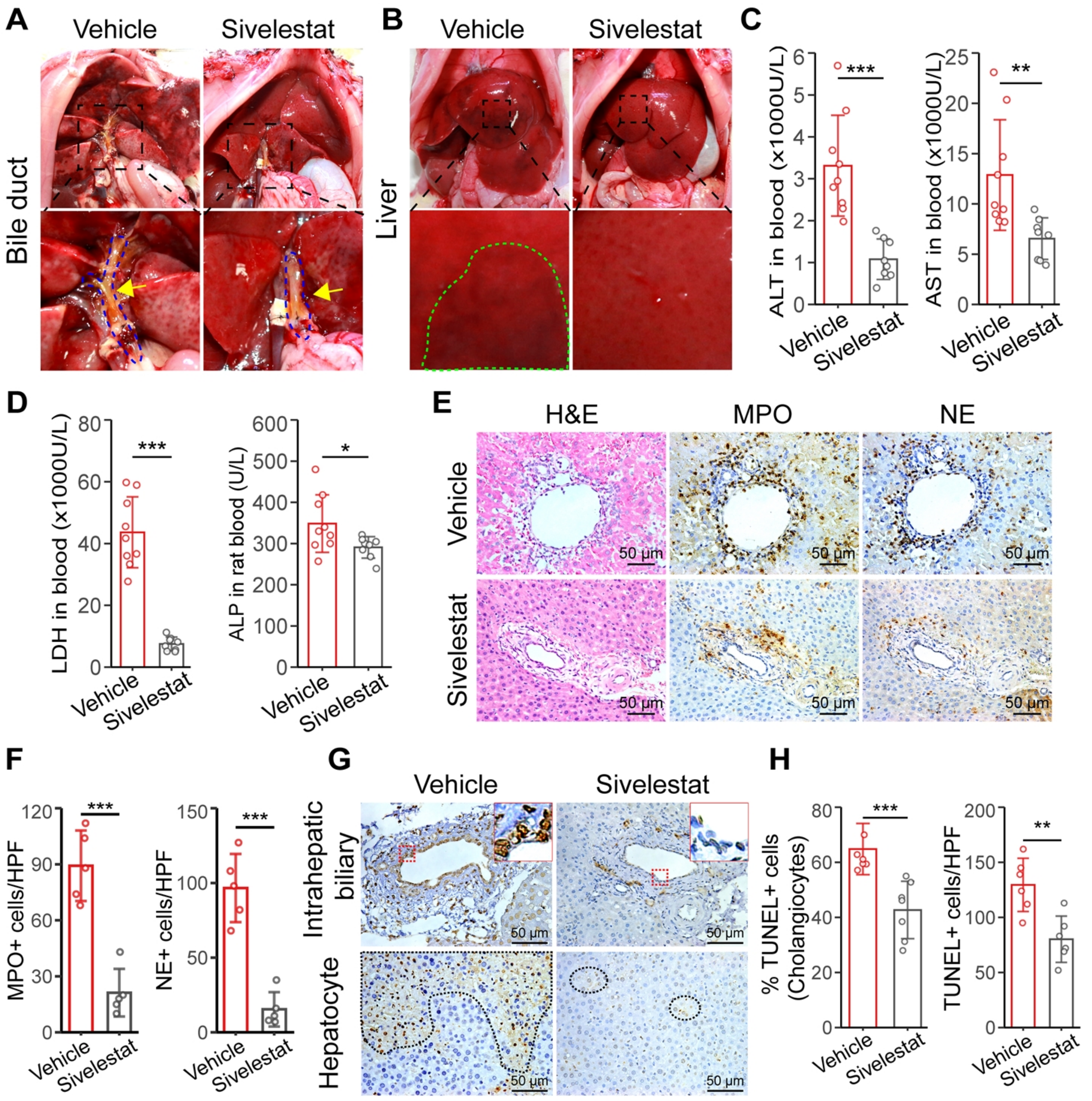

Figure S7. Images of liver anatomy in the extrahepatic bile duct **(A)** and liver **(B)** at 6 h post-surgery. The blue dashed line indicates the blue outline of the bile ducts, and yellow arrowheads show extrahepatic bile leakage, and the green outline indicates injury plaques. **(C, D)** Analyses of clinical parameters of hepatobiliary function in the peripheral blood of the vehicle group (n = 9) and sivelestat group (n = 8) at 6 h after surgery. **(E)** H&E staining and immunohistochemistry images of intrahepatic biliary injury and formed-NETs (indicated by MPO and NE). **(F)** Counting analysis of MPO- and NE-positive cells in the high powered field of image E (n = 5). **(G)** The TUNEL image of liver tissues from intrahepatic biliary cells and hepatocytes. The red dashed outline and black dashed outline signed biliary cells and hepatocytes, respectively. **(H)** Quantification of TUNEL-positive biliary cells percent and hepatic cells in image G, respectively (n = 6). Scale bar: 50 μm. Mean values ± SD. *p < 0.05; **p < 0.01; ***p < 0.001.

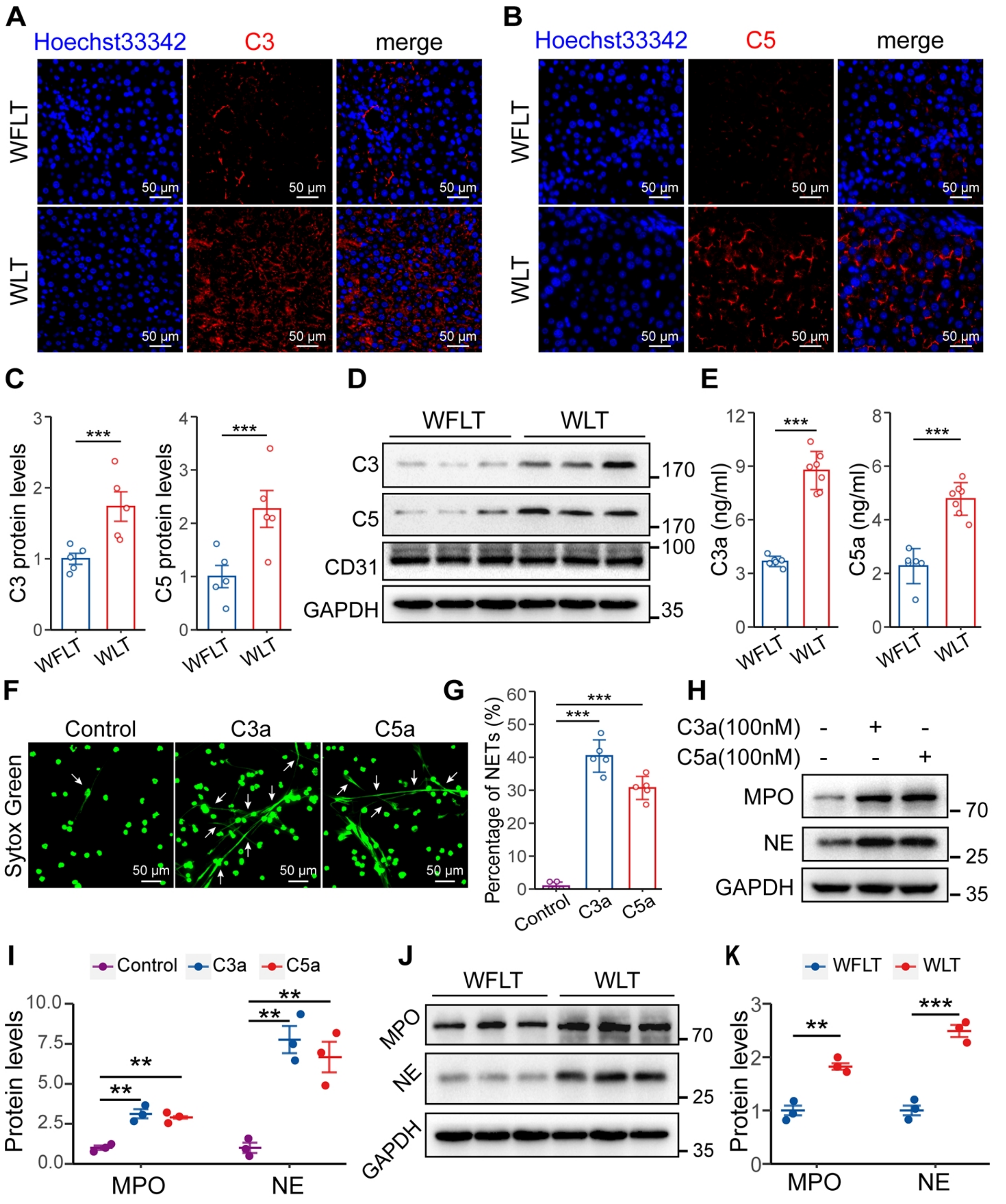

Figure S8. NET formation is closely related to complement C3 and C5 levels after liver transplantation. Immunofluorescence straining image of C3 **(A)** and C5 **(B)** in liver tissue samples at 6 h post-surgery. Blue indicates the cellular nucleus, and red indicates complement C3 or C5. **(C)** C3 and C5 protein levels in liver tissue determined by proteomics. **(D)** The protein levels of GPADH, CD31, C3 and C5 in grafted rat primary liver sinusoidal endothelial cells determined via western blotting. **(E)** The levels of C3a and C5a in the rat peripheral blood determined by ELISA (n = 6-7). **(F)** Immunofluorescence images of NET formation treated with 100 nM recombinant C3a or C5a for 6 h (n = 3), the green filamentous structure refers NETs (indicates by white arrow).

(G) Quantitative analysis of filamentous NETs in image F. **(H)** Western blotting image of GPADH, MPO, and NE in neutrophil cells stimulated with 100 nM recombinant C3a or C5a protein. **(I)** Quantitative analysis of normalization in image H (n = 3). **(J)** Western blotting image of GPADH, MPO, and NE in grafted primary neutrophil cells isolated from rat peripheral blood. **(K)** Quantitative analysis of normalization in image J. Scale bar: 50 μm. Mean values ± SD. **p < 0.01; ***p < 0.001.

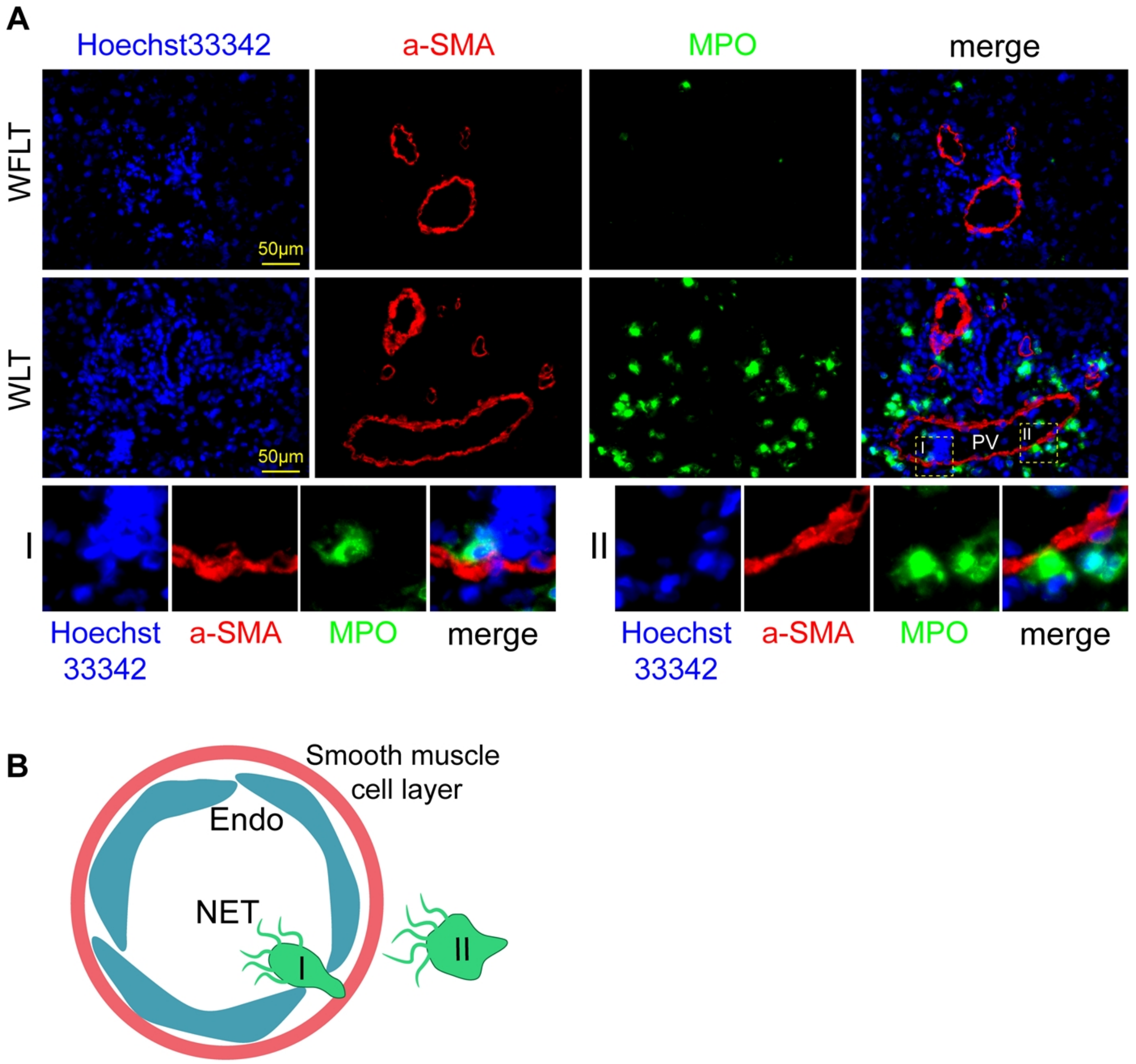

Figure S9. NET extravasation in tissue after liver transplantation. (A) Immunofluorescence staining of vascular smooth muscle (indicted by α-SMA) and NETs (indicated by MPO) in liver tissue samples. The blue labeled nucleus, and the white dashed line indicates the position of NETs. Scale bar: 50 μm. **(B)** Schematic illustration showing the criteria for delineating the two positions of NET extravasation in image A. PV, portal vein.

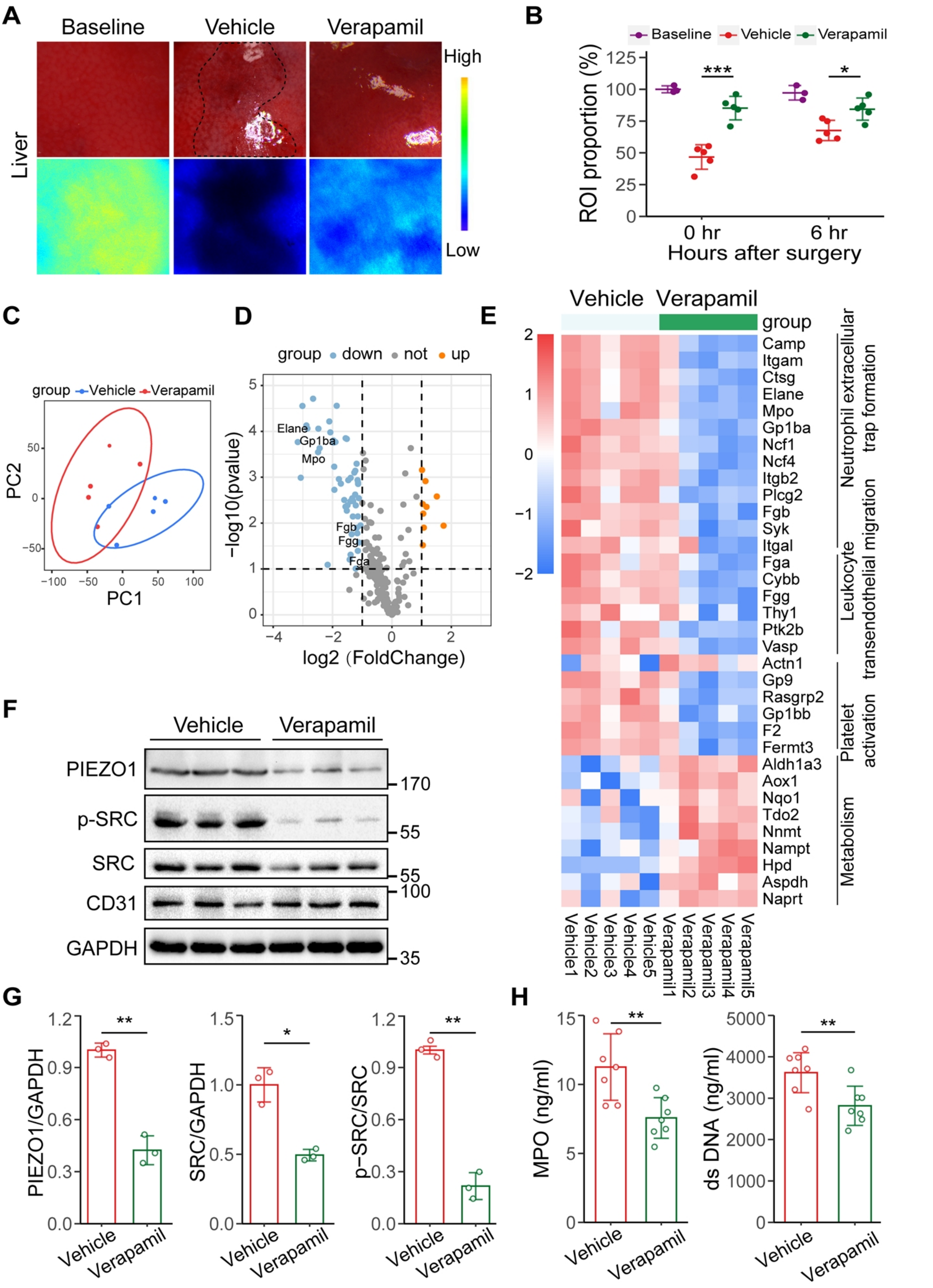

Figure S10. Intrahepatic blood flow and proteomic map in grafted rats pretreated with verapamil. (A) Laser

speckle images of hepatic blood flow at 0.25 h post-surgery, the black dashed outline indicates injured plaques. **(B)** ROI proportions of hepatic blood flow in baseline group (n = 3), vehicle group (n = 5) and verapamil group (n = 5) in the image A. **(C)** PCA plot of the vehicle and verapamil group. **(D)** Volcano plot of differential protein expression (absolute log2-fold change > 2, p value < 0.05) in NET formation, leukocyte transendothelial migration, platelet activation, and metabolism pathways. **(E)** Heatmap of pathway-related differential protein expression (absolute log2-fold change > 2, p value < 0.05). The red and blue color scales indicate upregulated and downregulated proteins, respectively. **(F)** Western blotting image of GPADH, CD31, PIEZO1, SRC, and p-SRC in verapamil-pretreated rat liver sinusoidal endothelial cells. **(G)** Quantitative analysis of normalization in image F. **(H)** The levels of MPO and dsDNA in the rat peripheral blood detected by ELISA and detection kit, respectively (n = 7). Mean values ± SD. **p < 0.05; **p < 0.01; ***p < 0.001.

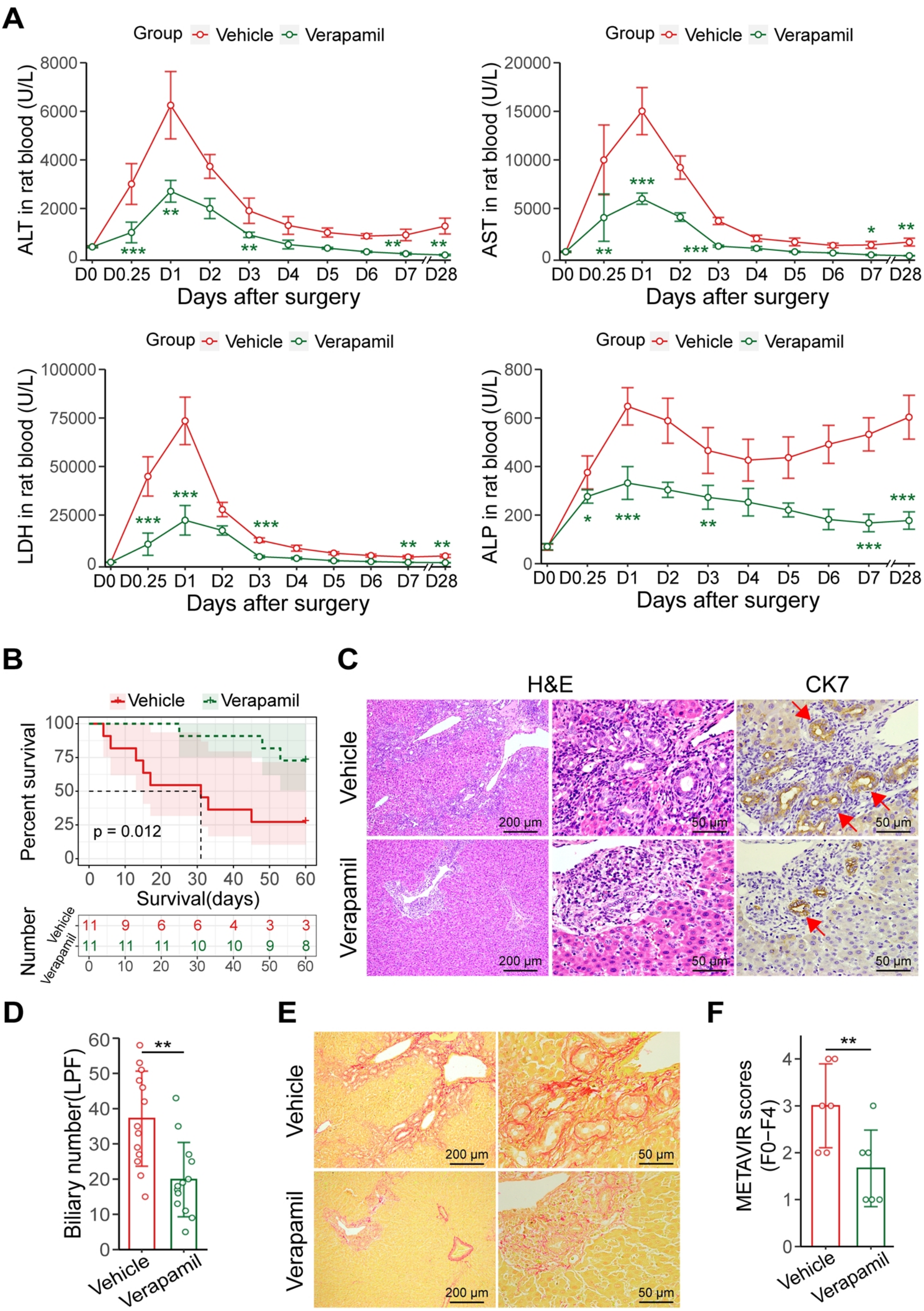

Figure S11. Biliary complications after liver transplantation pretreated with verapamil. (A) Analyses of

clinical parameters of hepatobiliary function in the peripheral blood of the vehicle group and verapamil group (n = 4-6 rat per time point). **(B)** Kaplan–Meier plot depicting the survival pattern of recipients in the vehicle group and verapamil group (n = 11). **(C)** Tissue section images of H&E and immunohistochemistry staining of grafted liver tissue from bile duct (indicated by CK7) after four weeks. **(D)** Quantification of CK7-positive cells of low powered field in image C. **(E)** Sirius Red staining images of grafted liver tissue after surgery. **(F)** Fibrosis scores of Sirius Red-positive areas in image E via METAVIR score. Scale bar: 200 µm and 50 µm. Mean values ± SD. * $p < 0.05$; ** $p < 0.01$; *** $p < 0.001$.
